# Synthesis, Characterization, and Evaluation of the Anxiolytic Activity of Hydrazones Derived from the Drug Isoniazid, Using the Adult Zebrafish (*Danio rerio*) Model

**DOI:** 10.1021/acsomega.5c04279

**Published:** 2025-09-25

**Authors:** Amanda Maria Barros Alves, Ivana Carneiro Romão, Rhadija Jorge Souza, Emmanuel Silva Marinho, Márcia Machado Marinho, Matheus Nunes Rocha, Kirley Marques Canuto, Jane Eire Silva Alencar Menezes, Sônia Maria Costa Siqueira, Hélcio Silva dos Santos

**Affiliations:** † 67843 Universidade Estadual do Ceará, Programa de Pós-Graduação em Ciências Naturais, Fortaleza, Ceará 60714-903, Brasil; ‡ Empresa Brasileira de Pesquisa Agropecuária, Embrapa, Fortaleza, Ceará 60511-110, Brasil

## Abstract

Anxiety, a multidimensional behavioral disorder, has been widely studied in neuroscience to understand its causes. The medications available for treatment show variable efficacy and side effects. To discover new drugs, animal models such as zebrafish (*Danio rerio*) have been used due to their genetic homology with humans. This study aimed to synthesize, characterize, and evaluate the anxiolytic activity of a hydrazone derivative in zebrafish models as well as to investigate its mechanism of action. The hydrazones were synthesized from the condensation of isoniazid with hydroxylated aldehydes and characterized by attenuated total reflectance Fourier transform infrared spectroscopy (ATR-FTIR) and NMR. For in vivo testing, six fish were treated with different doses of hydrazones (4, 20, 40 mg/kg), a negative control (3% DMSO), and a positive control (DZP 4 mg/kg), assessing locomotor activity and acute toxicity over 96 h. The light-dark test and neuromodulatory analysis of GABA and serotonin were also performed. The hydrazones (*E*)-*N*′-(2,4-dihydroxybenzylidene)­isonicotinohydrazide and (*E*)-*N*′-(2,3,4-dihydroxybenzylidene)­isonicotinohydrazide exhibited anxiolytic efficacy, reduced by flumazenil and granisetron. MPO analyses suggest that the compound resides in a physicochemical space formed by CNS-active drug candidates and exhibits ligand–receptor interactions, suggesting that the compounds may act similarly to the reference drug.

## Graphical abstract

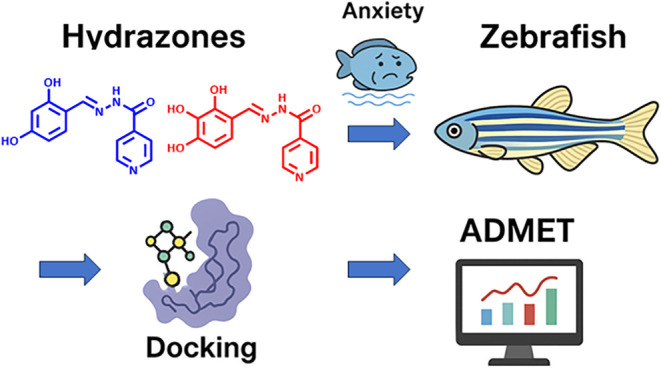

## Introduction

Although anxiety is a common emotional response, its persistence can evolve into a severe and debilitating psychiatric syndrome, resulting in a significant reduction in quality of life.^1^ Globally, anxiety is a prevalent psychiatric disorder that has a substantial impact on the overall functionality. For many years, benzodiazepines have been the primary approach to control anxiety, but their prolonged use is often associated with adverse side effects, including anterograde amnesia and psychological dependence, thus restricting their utility.^2^

The use of anxiolytics, such as selective serotonin reuptake inhibitors (SSRIs) and benzodiazepines (GABA receptor agonists), has limitations, such as sexual dysfunction, withdrawal syndrome, and considerable side effects.^3,4^ Anxiety that coexists with other conditions, such as depression and epilepsy, becomes even more challenging to control with existing treatments. These gaps in the approach to anxiety disorders highlight the ongoing need for research seeking new chemical substances with superior therapeutic profiles and more favorable side effects to improve treatment options for these disorders.

For the research of new drugs with anxiolytic properties, a variety of experimental animal models are available in the initial preclinical in vivo phase. One such model that has been gaining prominence is zebrafish (*Danio rerio*), especially in the fields of brain research and psychopharmacology. This vertebrate has become a significant model due to its genotype, which shares 70% exclusive homology with mammalian neurotransmitter receptors.^5^ Its notable characteristics, such as small size, high reproduction rate, rapid development, and transparency in early stages, considerably facilitate drug discovery in studies using this animal as a model.^6^ It is worth highlighting the relevance of using this animal model, as the literature reports studies on central nervous system (CNS) diseases in both natural products^7^ and synthetic products.^8^

Hydrazones are organic compounds commonly used as intermediates in organic synthesis and exhibit a variety of chemical and biological properties. They can be synthesized through condensation reactions between hydrazines and hydrazides with carbonyl compounds (aldehydes or ketones) in the presence of an acid or base catalyst.^9^ Thus, hydrazone compounds have drawn attention due to the presence of reported biological activities in the literature, such as in the treatment of anti-inflammatory and analgesic processes,^10^ diabetes,^11^ and central nervous system (CNS) diseases.^12^

In this context, this study aims to characterize and evaluate the anxiolytic activity of an *N*-acylhydrazone synthesized from the reaction of the drug isoniazid with 2,4-dihydroxybenzaldehyde and 2,3,4-dihydroxybenzaldehyde, in in vivo and in silico tests, using an adult zebrafish animal model.

## Results and Discussion

In this study, the objective was to conduct a comprehensive structural activity analysis of hydrazone derivatives, namely, HDZI 2,4OH and HDZI 2,3,4OH, and their properties involving acute toxicity and behavioral tests in different contexts. The primary aim was to evaluate the impact of these substances on aspects such as anxiety, pharmacokinetics, and molecular docking using adult zebrafish as the animal model (Figure 1).

**Figure 1:**
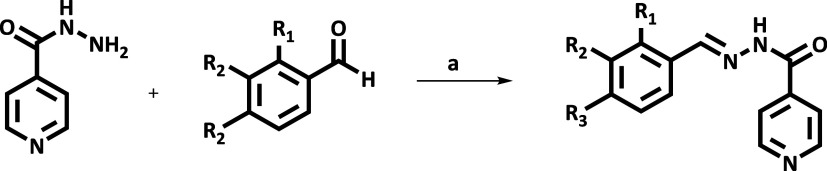
Representation of the synthesis of hydrazone.

### Structural Determination of Hidrazone (*E*)-*N*′-(2,4-Dihydroxybenzylidene)­isonicotinohydrazide and (*E*)-*N*′-(2,3,4-Trihydroxybenzylidene)­isonicotinohydrazide

The hydrazone (*E*)-*N*′-(2,4-dihydroxybenzylidene)­isonicotinohydrazide (HDZI 2,4OH) (Figure 2) has the molecular formula C_13_H_11_N_3_O_3_, and (*E*)-*N*′-(2,3,4-trihydroxybenzylidene)­isonicotinohydrazide (HDZI 2,3,4OH) (Figure 3) has the molecular formula C_13_H_11_N_3_O_4_; the identification of the obtained compound was performed using ^1^H and ^13^C NMR spectra analysis (1D and 2D) and comparison with previously published literature data.^12^ The ^13^C NMR data analysis shows that the product was formed by identifying the chemical shift value of the carbon atom attached to the nitrogen atom through a double bond (C=N), observed at δC = 149.1 ppm (HDZI 2,4OH) and 148.0 ppm (HDZI 2,3,4OH). The 1H shift values confirm the ^13^C data, supporting the formation of the hydrazone. Table 1 records the shift values for ^1^H and ^13^C NMR.

**Figure 2:**
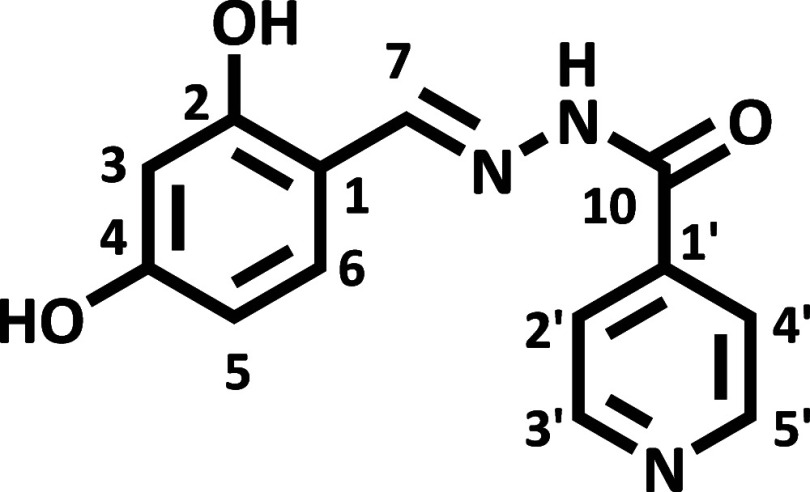
Structural representation of HDZI 2,4OH.

**Figure 3:**
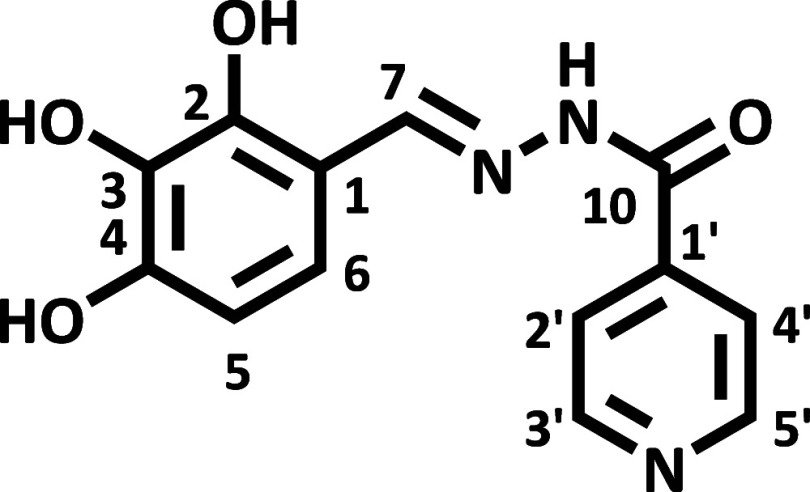
Structural representation of HDZI 2,3,4OH.

**Table 1:** NMR Spectroscopic Data for the Compound HDZI (^1^H: 500 MHz; ^13^C: 125 MHz; in DMSO)

	(*E*)-*N*′-(2,4-dihydroxybenzylidene)isonicotinohydrazide				(*E*)-*N*′-(2,3,4-trihydroxybenzylidene)isonicotinohydrazide			
	HSQC		HMBC		HSQC		HMBC	
	δ_C_	δ_H_	2JCH	3JCH	δ_C_	δ_H_	2JCH	3JCH
C								
1	111.6		H-6/H-7		111.1		H-6/H-7	
2	149.3				149.5			
4	149,1		H-5	H-6	150.7		H-5	H-6
1′	140.6		H-2′/H-6′	H-3′/H-5′	140.4		H-2′/H-6′	H-3′/H-5′
10	161.3			H-2′/H-6′	161.3			H-2′/H-6′
CH								
3								
5	108.7	7.00 (m)	H-6		108.2	6.89 (m)	H-6	
6	126.7	7.59 (d, *J* = 7.80 Hz)	H-5		133.7	7.80 (d, *J* = 7.80 Hz)	H-5	
2′/6′	121.3	7,84 (d, *J* = 4.85 Hz)			121.8	6.38 (d, *J* = 4.85 Hz)		
3′/5′	150.1	8.73 (d, *J* = 4,20 Hz)			151.5	6.81 (d, *J* = 4.20 Hz)		
7	149.1	8.72 (s)			148.0	11.25 (s)		
NH		9.00 (s)				12.13 (s)		
OH								

The infrared absorbance band observed between 3100 and 2900 in the attenuated total reflectance Fourier transform infrared (ATR-FTIR) spectrum is associated with the stretching modes of sp2 bonds (=CH). The bands present at 3442 cm^–1^ (HDZI 2,4OH) and 3279 cm^–1^ (HDZI 2,3,4OH) refer to N–H stretching, which is overlapped by hydroxyl (O–H) stretching. At 1681, 1622, and 1503 cm^–1^ for HDZI 2,4OH and at 1671, 1632, and 1551 cm^–1^ for HDZI 2,3,4OH, stretching bands corresponding to the amide carbonyl (C=O), the C=N–NH bond, and the aromatic ring C=C, respectively, can be observed, in addition to a band at 1291 cm^–1^ (HDZI 2,4OH) and 1243 cm^–1^ (HDZI 2,3,4OH) corresponding to the C–N bond,^13^ confirming the structure of the hydrazones compound.

### Acute Toxicity 96 h

The use of zebrafish to evaluate the toxicity of substances, especially during their embryonic and larval stages, is highly valuable. During the early development of this species, both cellular and molecular mechanisms are remarkably conserved compared to humans. This makes zebrafish a relevant experimental model for studying the toxic effects of various drugs and substances at early developmental stages.^14^ de Souza^12^ conducted a study with a hydrazone using the adult zebrafish model, which did not show toxicity. In an experiment guided by Popiolek,^15^ it was proven that hydrazone compounds and derivatives did not exhibit toxicity in tests conducted with rats. The hydrazone compounds did not show toxicity in adult zebrafish during the 96 h analysis (LD_50_ > 40 mg/mL).

### Assessment of Locomotor Activity (Open-Field Test—OFT)

The analysis of locomotor activity in adult zebrafish (*D. rerio*) is a common approach to evaluate the effects of drugs that affect the central nervous system and may cause behavioral changes.^16^ This analysis helps determine whether a substance can influence the locomotor behavior of the fish and can be conducted using open-field tests in aquariums or Petri dishes.^17^

In the open-field test, the animals are placed in an open environment such as an aquarium with appropriate lighting conditions. In this environment, the behavior of the fish is observed, and parameters such as the number of virtual line crossings and the distance traveled by the fish are measured, along with immobility, known as “freezing.” The natural behavior of zebrafish in an open environment is characterized by constant swimming activity, and immobility is rare under natural conditions. Therefore, the analysis of locomotor activity in an open field can serve as a model to evaluate swimming alterations, which may be associated with anxiety states in zebrafish.^17,18^

The number of line crossings in the open-field test showed that all three doses of the hydrazones and aldehydes reduced the locomotion of the animals (****p < 0.001 vs CONTROL; ^###^ *p* < 0.001, ^####^ *p* < 0.0001 vs DZP). These results were significantly different from those of the negative control (3% DMSO) (Figure 4).

**Figure 4:**
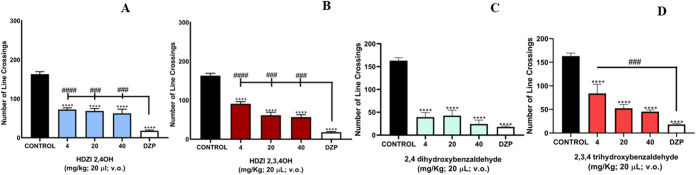
Effect of HDZI 2,4OH (A), HDZI 2,3,4OH (B), 2,4-dihydroxybenzaldehyde (C), and 2,3,4-trihydroxybenzaldehyde (D) on locomotor behavior of adult *zebrafish* in the open-field test (0–5 min). Each column represents the mean ± standard error of the mean. One-way ANOVA followed by Tukey’s test.

### Anxiolytic Assessment (Light/Dark Test—LDT)

The administration of anxiolytic drugs, such as benzodiazepines, to zebrafish can increase the exploratory activity of the fish in the open field, but they also have the potential to cause sedation and decrease locomotor activity. These studies provide valuable information on how different substances affect locomotor behavior and can help understand their impact on the central nervous system.^19,20^

The light-dark test showed that all three doses of the hydrazones, the three doses of 2,4-dihydroxybenzaldehyde, and the highest dose of 2,3,4-trihydroxybenzaldehyde (40 mg/kg) increased the time the animals spent in the light zone of the aquarium (*p < 0.05; **p < 0.01; ****p < 0.0001; Figure 5). These results were significantly similar to those of DZP (positive control) and different from those of the negative control (3% DMSO). Consequently, the mechanism of action of the lowest dose (4 mg/kg) of these compounds was evaluated.

**Figure 5:**
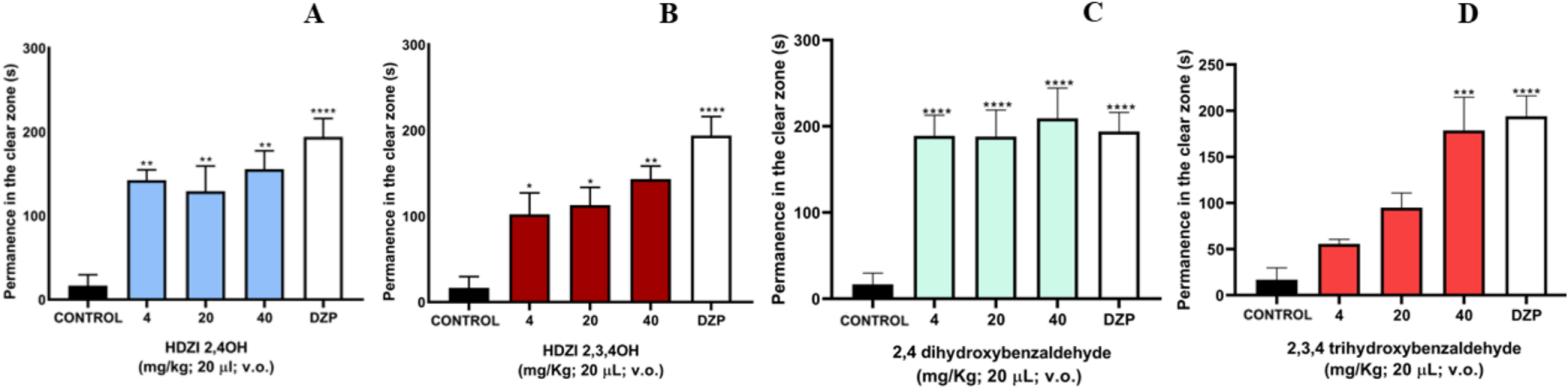
Effect of HDZI 2,4OH (A), HDZI 2,3,4OH (B), 2,4-dihydroxybenzaldehyde (C), and 2,3,4-trihydroxybenzaldehyde (D) on the anxiety behavior of adult *zebrafish* in the light and dark test (0–5 min). Each column represents the mean ± standard error of the mean. One-way ANOVA followed by Tukey’s test.

A study conducted with a hydrazone, aimed at evaluating the anxiolytic effect in adult zebrafish, showed a reduction in locomotor activity, yielding a result similar to this study.^12^ Conversely, tests conducted on rats to evaluate the analgesic effect of hydrazone compounds did not show changes in locomotion.^15^

The light-dark test is a commonly employed tool in neuroscience to assess the behavioral responses of animals to fear and anxiety. In this test, anxiolytic drugs are administered, which increase the time the fish spend in the illuminated area of an aquarium, while anxiogenic drugs have the opposite effect, decreasing the time in the illuminated area. These behavioral patterns are associated with the animal’s tendency to seek protection in the dark zone or to explore a new environment, choosing the illuminated zone^21^

### Assessment of GABAergic and SEROTONergic Neuromodulation

The mechanism of anxiety to evaluate the GABAergic pathway was assessed with pretreatment using flumazenil (FMZ), which showed that the activity of HDZI 2,4OH (4 mg/kg; ^####^ *p* < 0.0001 vs Treatment with FMZ) and 2,4-dihydroxybenzaldehyde (4 mg/kg; ^####^ *p* < 0.0001 vs Treatment with FMZ) was blocked by FMZ, an antagonist of the benzodiazepine binding site on GABAA receptors^22^ (S7).

Since HDZI 2,3,4OH and 2,3,4-trihydroxybenzaldehyde did not show anxiolytic effects via the GABAergic pathway, it was necessary to conduct tests to evaluate the serotonergic pathway. Pretreatment was performed with the antagonists cyproheptadine (5-HT2A), pizotifen (5-HT1 and 5-HT2*A*/2C), and granisetron (5-HT3A/3B). It was observed that the anxiolytic activity of HDZI 2,4OH (4 mg/kg; ^####^ *p* < 0.0001 vs treatment with antagonists) was blocked by all three pathways, indicating activity on 5-HT2A (cyproheptadine), 5-HT1 and 5-HT2*A*/2C (pizotifen), and 5-HT3*A*/3B (granisetron) receptors, HDZI 2,3,4OH (4 mg/kg; ^##^ *p* < 0.01; ^####^ *p* < 0.0001 vs treatment with antagonists) was blocked only after treatment with granisetron, indicating that the 5-HT3*A*/3B receptor corresponds to its pathway of action, 2,4-dihydroxybenzaldehyde (4 mg/kg; ^#^ *p* < 0.05; ^####^ *p* < 0.0001 vs treatment with antagonists) was blocked after treatment with cyproheptadine and granisetron, indicating activity through 5-HT2A and 5-HT3*A*/3B receptors, respectively. Finally, 2,3,4-trihydroxybenzaldehyde did not show anxiolytic effects via the serotonergic pathway, since none of the receptors tested blocked its activity (S8).

Anxiolytic compounds typically have hydroxyl substituents, nitrogen functions, halogens, nitro groups, methoxyl groups, and methyl groups in their structures.^23^ The structure of the studied hydrazone compound contains hydroxyl groups and nitrogen atoms, which may be associated with its anxiolytic activity, as reported in the literature. de Souza^12^ conducted a study to evaluate the anxiety response in adult zebrafish to a hydrazone compound, which showed an anxiolytic effect at its highest tested dose, acting via the GABAergic pathway. It is worth noting the lack of research on hydrazones related to anxiolytic effects.

Although the results obtained in this study demonstrate the anxiolytic potential of isoniazid derivatives in zebrafish, a complete elucidation of their mechanisms of action still requires further investigation. Considering that monoamine oxidase (MAO) plays a fundamental role in the metabolism of neurotransmitters, such as serotonin, dopamine, and norepinephrine, assessing the activity of this enzyme becomes a crucial step to determine whether the compounds act directly or indirectly on these systems. Changes in MAO activity can significantly impact synaptic monoamine levels, thereby modulating emotional and behavioral responses, including those related to anxiety.^43^

Therefore, future studies should include enzymatic assays to evaluate the potential inhibitory effects of these derivatives on the MAO-A and MAO-B isoforms as well as complementary neurochemical analyses that may establish correlations between the observed behavioral outcomes and possible alterations in monoamine metabolism. This approach will not only help clarify the underlying mechanisms but also enhance the translational relevance of the findings, since MAO modulators have well-established clinical applications in psychiatric disorders such as anxiety and depression.^44^ Thus, investigating MAO activity represents an essential step in advancing our knowledge about the pharmacological profile.

### MPO-Based Pharmacokinetics Prediction

In a topological analysis of HDZI, it is possible to observe that the structures 2,4OH (S9a) and 2,3,4OH (S9b) exhibit a structural increment of 0.65 in their relative lipophilicity, associated with the formation of an intramolecular H-bond interaction between the ortho–OH group and the H-bond accepting amine of the aliphatic structure of the compounds. It is possible to observe that HDZI 2,3,4OH presents a total of three OH groups of the HBD type and, for this reason, has a higher polarity than HDZI 2,4OH, resulting in a lower MLP surface (green color spectra). Despite having a similar hydrophobicity pattern in their aromatic structures (green to blue spectra), the H-bond donor (HBD) NH group contributes a highly polar region (red color spectra). For the precursors 2,4-dihydroxybenzaldehyde (S9c) and 2,4-trihydroxybenzaldehyde (S9d), the aldehyde carbonyl (C=O) presents a hydrophilic region, which, when combined with the polar surface of the OH groups, results in less lipophilic compounds, although they present lipophilicities similar to those of HDZI derivatives.

This analysis is corroborated by the logP value of 1.48 for the 2,3,4OH-substituted compound compared to its 2,4OH analogue (log *P* = 1.79), whose polarity resulting from the HBD groups falls outside the desirability spectrum (S9f), resulting in an MPO score of 4.17, and thus below the ideal activity spectrum for CNS (MPO < 5). In contrast, HDZI 2,4OH has an MPO score of 5.09 (Table 2).

**Table 2:** Physicochemical Properties Applied to the Pfizer, Inc., Druglikeness Classification System, and In Vitro ADME Attributes Predicted by In Silico Pharmacokinetics from the ADMETlab 2.0 and ADMET–LMC Servers

property	HDZI 2,4OH	HDZI 2,3,4OH	2,4diOH	2,3,4triOH	ISOZD	Optimal
Medicinal Chemistry Properties						
log *P*	1.79	1.48	1.73	1.43	0.53	≤3.0
log *D*7.4	1.78	1.46	1.55	1.29	0.53	≤2.0
MW	**257.25 g/mol**	**273.25g/mol**	138.12 g/mol	154.12 g/mol	136.15 g/mol	200–500
TPSA	94.91 Å^2^	115.04 Å^2^	57.53 Å^2^	77.76 Å^2^	55.12 Å^2^	40–120
HBD	3	4	2	3	3	≤1
p*K*_a_ most basic	3.03	3.03	–6.07	–6.40	2.74	≤8.0
MPO score	**5.09**	4.17	**5.50**	**5.25**	**5.25**	≥4.0
Pfizer rules	Accepted	Accepted	Accepted	Accepted	Accepted	
ADME Properties						
Papp, A → B	**9.3 × 10** ^**–6**^ **cm/s**	7.6 × 10^–6^ cm/s	8.1 × 10^–6^ cm/s	5.3 × 10^–6^ cm/s	**3.8 × 10** ^**–5**^ **cm/s**	≥10 × 10^–6^
P-gp efflux	0.24	0.29	0.01	0.00	0.01	≤0.25
VD	0.69 L/kg	0.43 L/kg	0.84 L/kg	0.53 L/kg	0.65 L/kg	≤1.0
CL_int,u_	**4.96** mL/min/kg	**3.54** mL/min/kg	12.94 mL/min/kg	12.10 mL/min/kg	**7.13** mL/min/kg	≤8.0
HIA	**98.67%**	51.58%	**90.62%**	65.86%	**100%**	≥75
log(*C*_brain_/*C*_blood_)	**0.30**	–0.58	–0.37	–0.7	0.22	≥0.30

To facilitate the process of selecting safe CNS drugs, a machine-learning-assisted MPO algorithm implemented by Pfizer, Inc., can predict the target profile of a drug candidate, aiming to meet unmet needs beyond the limitations of the Rule of Five and Leadlikeness. According to Wager,^24^ weak bases that are minimally lipophilic (log *P* ≤ 3) and larger and more polar than active CNS drugs (TPSA 20–120 Å^2^) show a better alignment between in vivo toxicity and in vitro ADME attributes, which include high passive cellular permeability (Papp, A → B > 10 × 10^–6^ cm/s), low incidence of P-gp efflux, and low hepatic clearance rates (CL_int,u_ < 8.0 mL/min/kg).^25^ These attributes indicate high cell viability (Cv) and low CNS toxicity incidence.^26^ When aligned, these attributes are excellent parameters for selecting drugs with good oral bioavailability and low toxicity, corresponding to about 50% of failures in clinical trials (van der Waterbeemd and Gifford, 2003).

### ADME Descriptors

The empirical decisions of MPO analyses align with the predicted pharmacokinetic descriptors from the ADME test. The analyses revealed that the HDZI 2,4OH and ISOZD compounds had the best fit within the favorable physicochemical space for CNS candidates, characterized by log *P* < 3 and TPSA > 75 Å^2^,^26^ indicating a structural and pharmacological similarity (S10a). Within a range of MW < 360 g/mol, the lipophilicity at physiological pH (log *D*7.4) of the compounds between 1 and 2 suggests a balance between oral absorption and metabolic stability (S10b), attributes strongly associated with the predicted descriptors of Papp, A → B at the order of 9.3 × 10^–6^ cm/s and the CL_int,u_ rate at 4.96 mL/min/kg for the HDZI 2,4OH ligand. On the other hand, the precursors 2,4-dihydroxybenzaldehyde and 2,3,4-trihydroxybenzaldehyde present MW < 200 g/mol, shifting toward the ideal pharmacokinetic spectrum, which may reduce their metabolic stabilities in first-pass metabolism (S10b).

These values indicate, respectively, considerable permeability (though below the ideal limit) combined with low hepatic clearance.^27^ Such pharmacokinetics are associated with an estimated HIA of 98.67%, contrary to HDZI 2,3,4OH, where Papp, A → B < 8.0 × 10^–6^ cm/s resulted in low predicted gastrointestinal tract absorption (S10c), a feature strongly associated with the aryl substructure containing 3 OH groups.^28^ Additionally, the TPSA value of 94.91 Å^2^ favors the permeability of HDZI 2,4OH across the blood-brain barrier (BBB) compared to HDZI 2,3,4OH, with a permeability coefficient based on the brain-blood distribution ratio (Cbrain/Cblood) of 0.3,^29^ indicating that the free fraction in the blood readily penetrates the CNS (S10d). On the other hand, the BBB permeability of the precursors 2,4-dihydroxybenzaldehyde and 2,3,4-trihydroxybenzaldehyde is affected by their physicochemical attributes, which may be strongly related to low stability in first-pass metabolism, with logBB (Cbrain/Cblood) calculated in the order of −0.37 and −0.7, respectively (S10f).

Being a more lipophilic compound, HDZI 2,4OH is favored in its distribution to tissues. The predicted VD value of 0.69 L/kg suggests that the hydrophobic character of HDZI 2,4OH promotes a distribution balance shifted toward the organic phase of the human physiological system, including biological tissues, enhancing its permeation in different physiological compartments, especially the BBB.^30^ Furthermore, CL_int,u_ values less than 8.0 mL/min/kg are observed for the compounds HDZI 2,4OH and HDZI 2,3,4OH, which have been shown to be more metabolically stable than the precursors 2,4-dihydroxybenzaldehyde and 2,3,4-trihydroxybenzaldehyde and the compound ISOZD, according to Pfizer’s classification system.^25^ This suggests that they possess favorable oral bioavailability and low reactivity to first-pass metabolism (Table 2).

### Site of Metabolism Prediction

The prediction of the site of metabolism allows us to estimate the toxic risk of new drug candidates based on their chemical structure. Molecular fragments that are substrates for CYP450 can be potentially toxic due to metabolic activation. For example, epoxides, which are intermediates in aromatic hydroxylation reactions, have electrophilic potential and can interact with macromolecules such as proteins and DNA or form glutathione-based conjugates.^26^

In the sensitivity map, based on the specificity of functional groups for CYP450 isoforms, it is observed that the amine group on the heteroaromatic ring, for both HDZI compounds, shows a probability of around 0.6 for biotransformation via N-oxidation by the CYP3A4 isoform (S11). However, the p–OH group experiences a greater electron-withdrawing inductive effect from the carbonyl and is more susceptible to O-conjugation (green color spectra), as can be seen in the chemical structures of HDZI 2,4OH (S11a) and HDZI 2,3,4OH (S11b), by the enzyme UDP-glucuronosyltransferase (UGT) during phase II metabolism. This makes the substance resistant to presystemic metabolism (phase I) and, by extension, less reactive to epoxidation of its aromatic centers. The compounds demonstrated greater similarity with low-reactivity molecular fragments, within a threshold >0.7, indicating low toxicity from metabolic activation, such as acute oral toxicity.

On the other hand, the precursors 2,4-dihydroxybenzaldehyde and 2,3,4-trihydroxybenzaldehyde present an aldehyde group (CHO) that can undergo CYP3A4- and CYP2D6-dependent reduction in phase I metabolism, indicating that they can form epoxidized radicals, which can be reactive to the human liver by forming undesirable drug interactions (S11c,d), with a high degree of similarity with molecular fragments deposited in the STopTox database (>0.8), while ISOZD presents a low structural specificity in the prediction of the metabolism site (outside of the threshold) (S11e).

### Structure-Based Virtual Screening

According to Wager,^24^ compounds with a higher cell viability (Cv), resulting from an alignment between high Papp and low CL_int,u_, also showed high affinity for G-protein-coupled receptors (GPCRs), ion channels, and enzymes, particularly CNS-active drugs with an MPO score >5. It is noteworthy that HDZI 2,4OH exhibited structural similarity to 18 bioactive compounds that modulate GABAA receptors in the CNS, mainly due to the aliphatic amide substructure linking two aromatic systems. On the other hand, HDZI 2,3,4OH showed affinity with at least 11 compounds binding to 5-HT receptor types, but with less specific pharmacophores.

Here, it was observed that HDZI 2,4OH presented an MPO score >5 and showed, through structure-based virtual screening, about 26.6% of its biological interactions with GPCRs, 13.4% with ion channels, and 13.3% as a substrate for biological enzymes, such as transporters and metabolic enzymes (Figure 6a). In contrast, HDZI 2,3,4OH predominantly targeted kinases in its pharmacodynamics (26.7%), though it also had affinities with enzymes (33.3%) and GPCRs (13.4%), but showed low specificity for ion channels (Figure 6b), scoring an MPO < 5 (Table 2).

**Figure 6:**
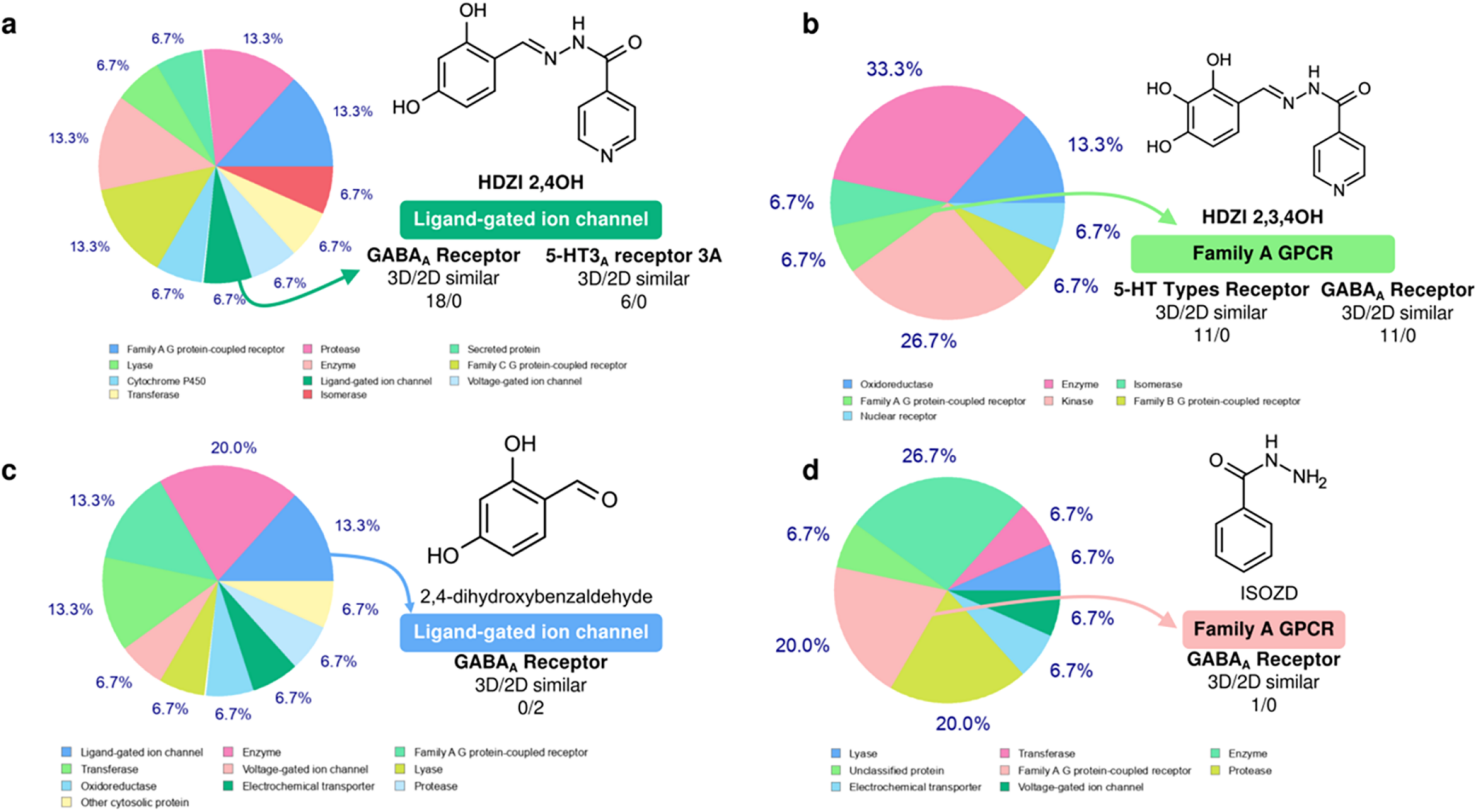
Structure-based virtual screening of target class prediction of (a) HDZI 2,4OH, (b) HDZI 2,3,4OH, (c) 2,4-dihydroxybenzaldehyde, and (d) ISOZD. 2,3,4-Trihydroxybenzaldehyde was omitted because it did not show similar bioactivity against 5-HT_3A_ and GABA_A_ receptors in the predictive test.

It is noteworthy that HDZI 2,4OH showed structural similarity with 18 bioactive compounds that modulate GABAA receptors in the CNS, particularly due to the aliphatic amide substructure linking two aromatic systems (Figure 6a), although it showed similarity with 5-HT_3A_ modulators. On the other hand, HDZI 2,3,4OH exhibited affinity with at least 11 ligands of 5-HT receptor types, and 11 similar GABA_A_ modulators, though with less specific pharmacophores (Figure 6b). For the HDZI derivative precursors, it was observed that 2,4-dihydroxybenzaldehyde showed 2D similarity to only two GABA_A_ receptor modulators, while 2,3,4-trihydroxybenzaldehyde showed no similarity to bioactive compounds acting on either of the two receptors (5-HT_3A_ and GABA_A_) (Figure 6c). ISOZD, on the other hand, showed similarity to only one GABA_A_ modulating ligand identified in the ChEMBL database (Figure 6d).

These results suggest that structural modifications can promote more efficient analogues targeting GABA_A_ and 5-HT_3A_ receptors, but the synthetic HDZI precursors 2,4OH and 2,3,4OH exhibit low structural specificity for these pathways.

### Binding Analysis in GABAA and 5-HT3A Receptors

The theoretical anxiolytic mechanism was investigated through molecular docking simulations targeting 5-HT3A and GABAA receptors. At the end of the cycle of independent molecular docking simulations, the best pose was chosen from a ranking by affinity energy (*E*_A_), filtered by the statistical parameter of root-mean-square deviation (RMSD) ≤ 2.0 Å.^31^ With the results, it was possible to observe that all the chosen best poses performed within a reliable statistical threshold (RMSD ≤ 2.0 Å), where E_A_ lower than ≤−6.0 kcal/mol is the energetic parameter that expresses a good binding affinity of the compounds.

Nitrogenous bases such as hydrazones and hydrazines have shown promising effects against anxiety and depression, associated with mechanisms of action such as the modulation of 5-HT receptor types and even GABAA receptors.^12^ This has sparked significant interest among researchers in these pharmacophores. In this context, the goal is to identify new anxiolytic agents derived from commercial drugs, such as Apresoline and Isoniazid, that exhibit an affinity for these receptor classes. Furthermore, the molecular docking simulations performed here consider the protein to be rigid and the ligands to be flexible, disregarding the flexibility of the amino acid residues that make up the binding sites. Furthermore, contributions from the solvent environment (CNS), which are generally more sensitive to solvation in molecular dynamics simulations, were not considered in this study.^31^ This indicates that the affinities analyzed refer to the steady state of the formed ligand–receptor complex.

Corroborating the structure-based virtual screening process and after completing 50 independent cycles of 20 poses each (for each ligand), it was observed that HDZI 2,4OH achieved a better binding affinity (−6.995 kcal/mol) compared to the drug ISOZD (−5.626 kcal/mol), but with higher energy when compared to the analogue HDZI 2,3,4OH, with the affinity energy (*E*_A_) calculated at −7.646 kcal/mol (Figure 7a). However, the inhibitor DZP demonstrated the highest affinity among the simulations with the GABAA receptor, with a binding energy of −8.411 kcal/mol (Figure 7a), indicating that HDZI 2,4OH and HDZI 2,3,4OH have strong potential as a GABAA receptor modulator (Shityakov and Foerster, 2014), although they showed a lower affinity spectrum than the agonist. On the other hand, HDZI 2,4OH and HDZI 2,3,4OH bound to the 5-HT3A receptor with a E_A_ of −8.092 and −7.694 kcal/mol, respectively (Figure 7b), but showed lower affinity compared to the calculated values for the comparative drug Fluoxetine (−8.352 kcal/mol) and the inhibitor CBW (−9.516 kcal/mol), although the binding affinities were within the ideal range (<−6.0 kcal/mol).

**Figure 7:**
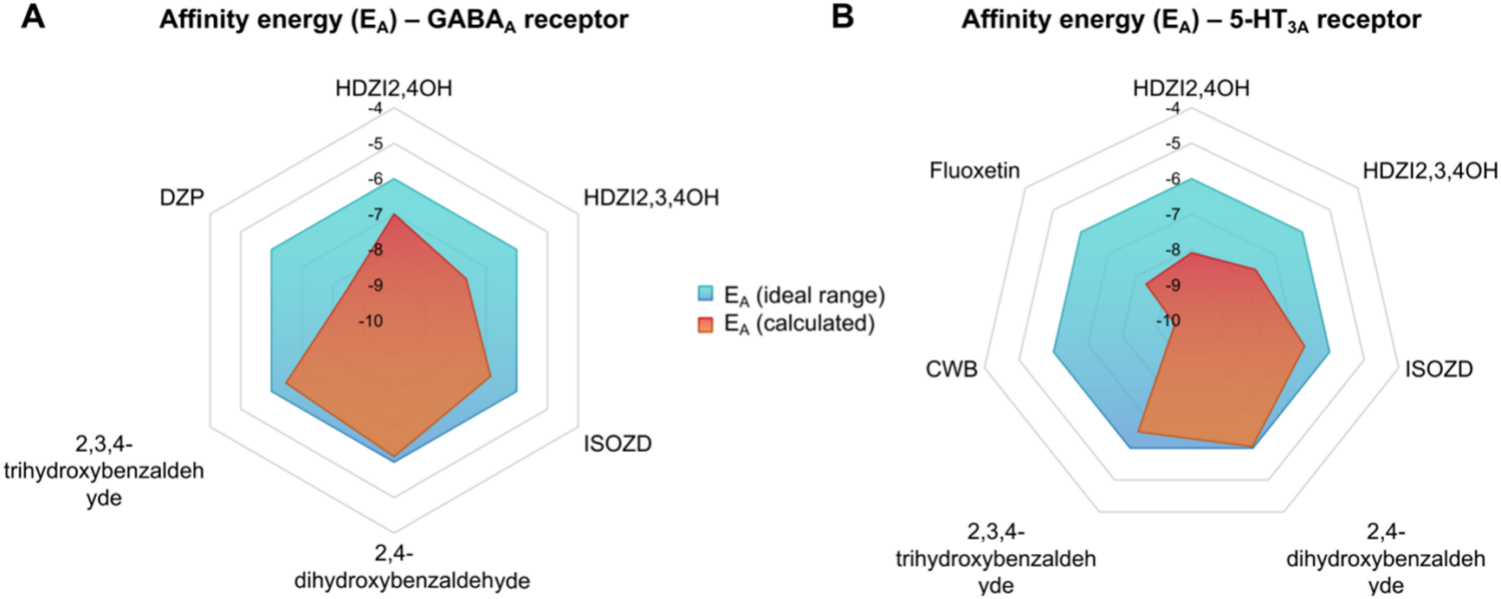
Affinity energy values of HDZI analogues toward GABAergic and serotonergic systems in relation to control ligands (inhibitors) and reference drugs.

It is interesting to note that the ISOZD precursors, 2,4-dihydroxybenzaldehyde and 2,3,4-trihydroxybenzaldehyde, interacted with GABAA and 5-HT3A receptors with calculated *E*_A_ > 6.8 kcal/mol, indicating that they present lower specificity for the receptor binding sites than the synthesized HDZI analogues (Figure 7).

When analyzing the docking poses with the GABAA receptor, it was observed that HDZI 2,4OH and HDZI 2,3,4OH bind to the DZP site located in the extracellular domain between the α1-D and γ2-C chains (Figure 8a,b), indicating that the compounds compete for the DZP binding site (Figure 8c). Regarding the precursors, the 2,4-dihydroxybenzaldehyde fragment showed greater specificity for the benzodiazepine binding site when compared to 2,3,4-trihydroxybenzaldehyde (Figure 8d).

**Figure 8:**
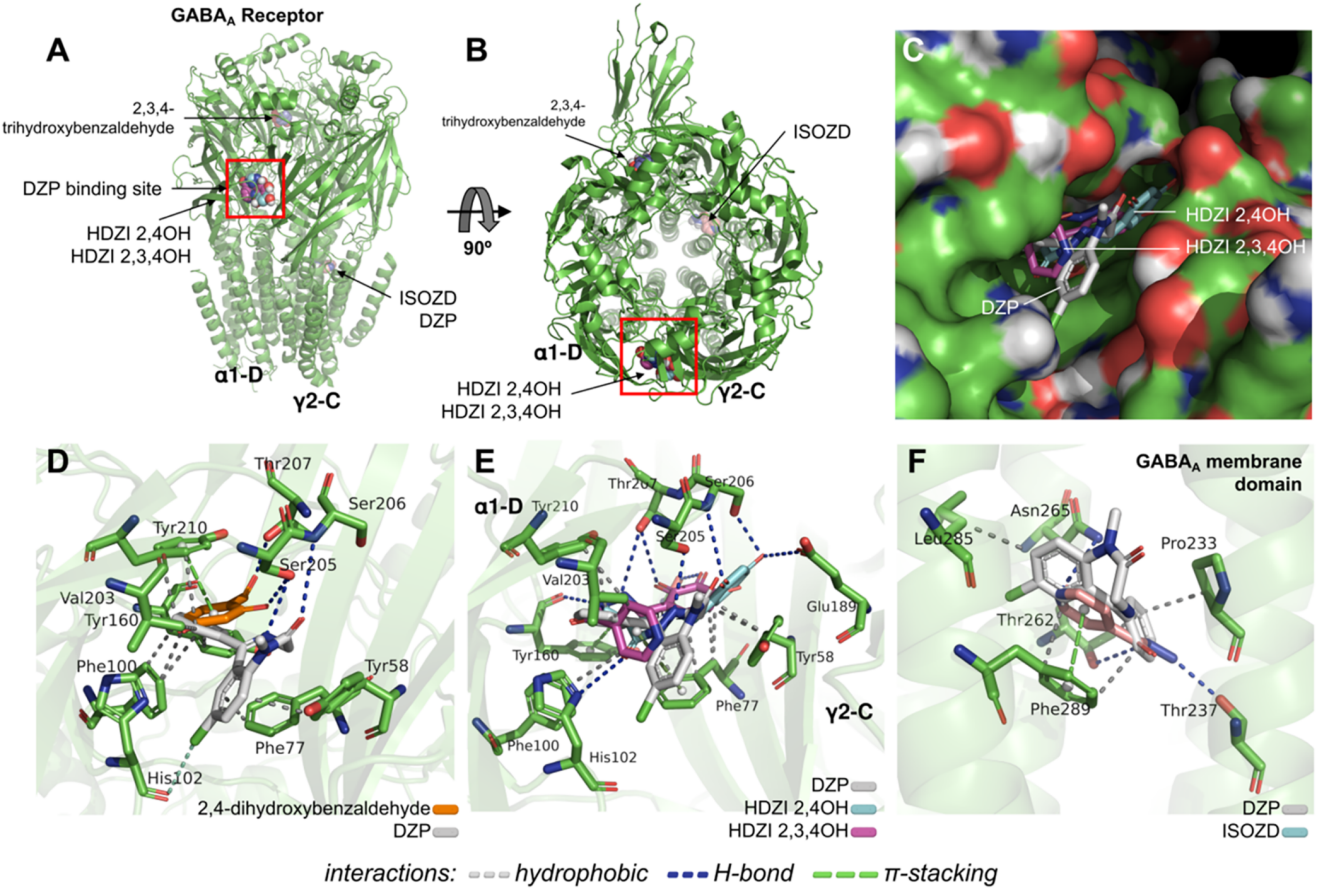
(a) Three-dimensional perspective and (b) 90° view of the docking of HDZI 2,4OH and HDZI 2,3,4OH with respect to the diazepam (DZP) binding sites and the GABAA receptor binding domains. (c) Superposition view of HDZI 2,4OH and HDZI 2,3,4OH and the agonist DZP in the binding pocket of the extracellular binding domain. Three-dimensional view of ligand–receptor interactions comparing (d) 2,3,4-trihydroxybenzaldehyde and DZP, (e) HDZI 2,4OH, HDZI 2,3,4OH and DZP, and (f) ISOZD and DZP.

HDZI 2,4OH and HDZI 2,3,4OH share interactions with the benzodiazepine binding site residues, including Tyr160D, Ser206D, Ser205D, His102D, Tyr210D, Phe77C, and Tyr58C (Figure 8e). This interaction has an RMSD value of 1.81 Å, indicating excellent statistical specificity of the ligand for the binding site. The ligands showed RMSD values <2.0 Å at the end of simulations. ISOZD binds to the DZP site located in the membrane domain between the α1-D and β3-E chains (Figure 7c), suggesting it may act synergistically with HDZI 2,4OH or HDZI 2,3,4OH in the allosteric modulation of the GABAA receptor.^32^

Interestingly, the aromatic centers of HDZI 2,4OH and HDZI 2,3,4OH contribute significantly to the formation of hydrophobic interactions in common with DZP, such as the pyridine ring of the compound with the aromatic centers of Tyr160D and Tyr210D residues and the diphenol ring with the aromatic residue Tyr58C (Figure 8e). Additionally, a moderate-strength H-bond interaction (*d* = 2.31 Å) is formed with the Ser206D residue via the p–OH group of the HDZI 2,4OH^33^ (Figure 8e). ISOZD showed greater specificity for residues in the DZP binding site located in the transmembrane domain, indicating a synergistic effect with drugs that bind to the extracellular domain of the GABAA receptor (Figure 8f).

Regarding the 5-HT3A target, it was possible to observe that the HDZI 2,4OH and HDZI 2,3,4OH analogues complexed to the CWB site (Figure 9a) located between the principal and complementary subunits (Figure 9b), suggesting a modulation by competition for the same binding site in the extracellular domain of the 5-HT3A receptor (Figure 9c). This binding shares interactions with residues Tyr207E, Ile201E, and Trp63D relative to the inhibitor. Fluoxetine (control-drug) interacted with a distinct site where CBW was also cocrystallized, forming primarily hydrophobic interactions with aromatic residues Trp63A, Tyr126A, Trp156B, and Trp63D (Figure 9d). Meanwhile, the ligands showed RMSD values <2.0 Å at the end of the simulation cycle, indicating good specificity for their respective binding sites.

**Figure 9:**
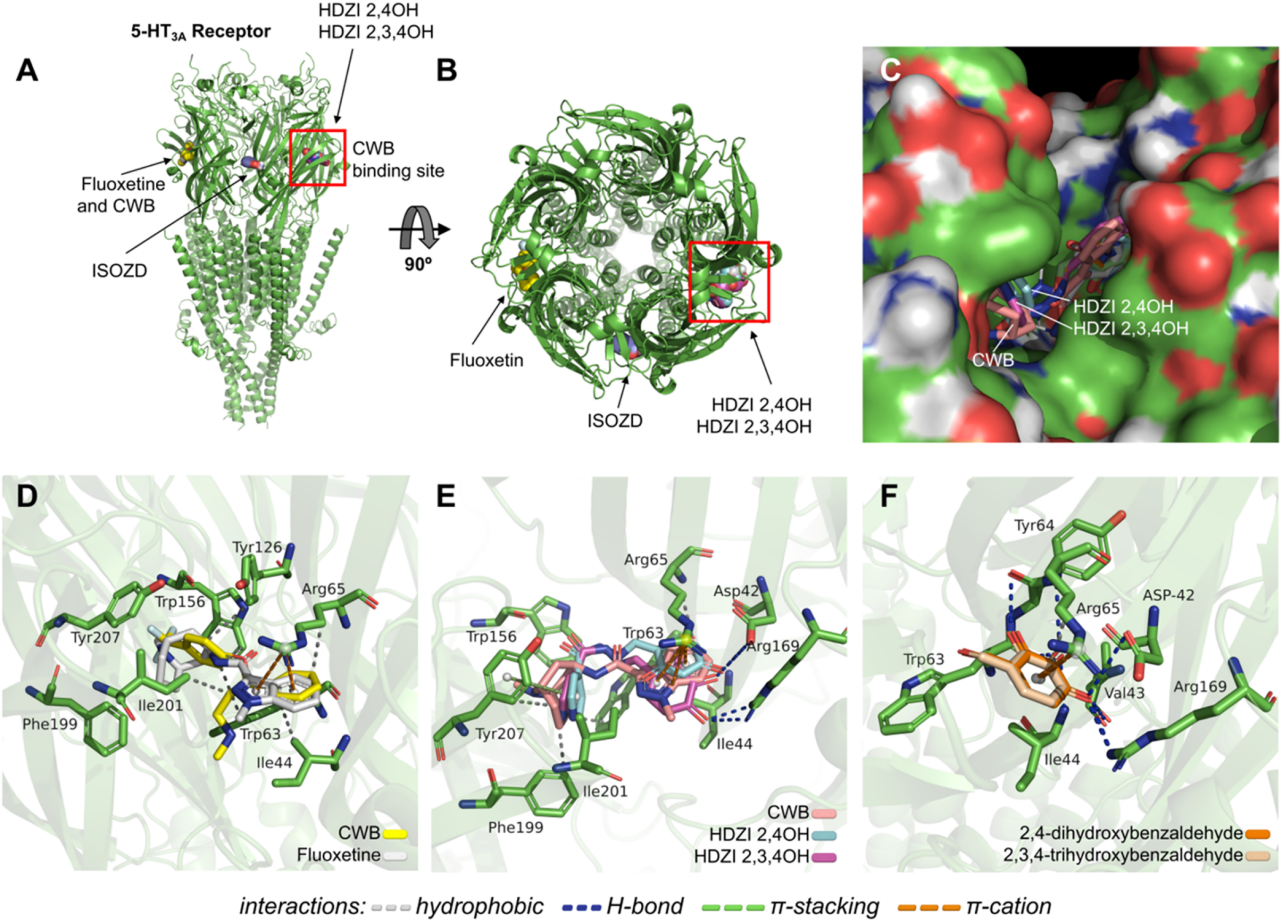
(a) Three-dimensional perspective and (b) 90° view of the docking of HDZI 2,4OH and HDZI 2,3,4OH with respect to the CWB binding sites and the 5-HT3A receptor binding domains. (c) Overlap between the ligands HDZI 2,4OH and HDZI 2,3,4OH and the inhibitor CWB in the extracellular binding domain of the 5-HT3A receptor. Three-dimensional view of ligand–receptor interactions comparing (d) fluoxetin and CWB, (e) HDZI 2,4OH, HDZI 2,3,4OH and CWB, and (f) 2,4-dihydroxybenzaldehyde and 2,3,4-trihydroxybenzaldehyde.

The HDZI 2,4OH and HDZI 2,3,4OH compounds share interactions with residues Tyr207E, Ile201E, and Trp63D relative to the inhibitor (Figure 8e), while their substituted aromatic rings formed π-cation interactions with the positively charged portion of the Arg65 residue (Figure 9e), indicating that they present high specificity for this binding site. This corroborates the simulations performed with the precursors 2,4-dihydroxybenzaldehyde and 2,3,4-trihydroxybenzaldehyde, which share interactions in common with the HDZI derivatives (Figure 9f). Meanwhile, Fluoxetine interacted with a distinct site where CBW was also cocrystallized (Basak et al., 2019), forming primarily hydrophobic interactions with aromatic residues Trp63A, Tyr126A, Trp156B, and Trp63D (Figure 8c). This suggests that HDZI 2,4OH and HDZI 2,3,4OH are strong candidates for modulating the 5-HT3A receptor, potentially acting synergistically with the drug Fluoxetine.

## Conclusions

The spectroscopic analysis provided information about the structural, vibrational, and electronic properties of the hydrazones. Regarding anxiolytic activity, the data revealed that the hydrazones caused alterations in the locomotor system of zebrafish without showing toxicity over 96 h. Treatments with lower doses resulted in anxiolytic behavior in the animals. This effect was reduced by the administration of flumazenil, suggesting that the anxiolytic activity of synthesized HDZI 2,4OH occurs through modulation of the GABAergic system, while HDZI 2,3,4OH showed reduced effects after administration of granisetron, a serotoninergic system antagonist acting via the 5-HT3*A*/3B pathway. These findings highlight the relevance of hydrazone compounds as potential candidates for the development of new anxiolytic drugs. MPO analyses suggest that these compounds reside in a physicochemical space aligned with active CNS drug candidates, characterized by a balance between Cv and metabolic stability, especially due to low lipophilicity and high polarity. Structure-based virtual screening indicates that they are potentially modulatory compounds of ion channels and GPCRs, corroborating in vivo tests and molecular docking simulations. Here, a synergistic pathway of action is proposed for HDZI 2,4OH on the GABAA receptor as well as for HDZI 2,3,4OH associated with Fluoxetine on the 5-HT3A receptor, where affinity energy and ligand–receptor interactions suggest they are strong candidates for drugs acting on these pathways in anxiety treatment.

## Experimental Section

### Drugs and Reagents

The following substances were used: diazepam (DZP, Neo Qumica), flumazenil (Fmz; Sandoz), dimethyl sulfoxide (3% DMSO; Dynamic), 2,4-dihydroxybenzaldehyde (Sigma-Aldrich), 2,3,4-trihydroxybenzaldehyde (Sigma-Aldrich), phosphoric acid (Sigma-Aldrich), sodium bicarbonate (Sigma-Aldrich), fluoxetine (Sandoz), and ethanol (Sigma-Aldrich).

### Synthesis and Characterization of Hydrazones Derivatives

#### Synthesis (*E*)-*N*′-(2,4-Dihydroxybenzylidene)­isonicotinohydrazide (HDZI 2,4OH) and (*E*)-*N*′-(2,3,4-Trihydroxybenzylidene)­isonicotinohydrazide (HDZI 2,3,4OH)

Hydrazones were synthesized through an acid-mediated reaction. In a 25 mL reaction flask, 0.50 mmol of 2,4-dihydroxybenzaldehyde (for HDZI 2,4OH) and trihydroxybenzaldehyde (for HDZI 2,3,4OH), 0.50 mmol of the drug isoniazid, 9.0 mL of distilled water, and 1.0 mL of concentrated H3PO4 were mixed. This reaction mixture was subjected to magnetic stirring with heating at 100 °C for 45 min. Subsequently, 15 mL of absolute ethanol was added to the reaction mixture, which was then filtered, and the filtrate was collected in a beaker. The residue retained on the filter paper was discarded. To the filtrate, 20 mL of an ice-cold aqueous solution of NaHCO3 (5.0% w/v) was added. The resulting solid was vacuum-filtered, washed with ice-cold absolute ethanol, and dried in an oven at 75 °C for 30 min. After cooling, the solid was removed from the filter paper and weighed.^9^

### Spectroscopic Methods: NMR, FTIR

^1^H and ^13^C NMR spectra were obtained using a Bruker DRX 500 MHz, operating at a frequency of 500 MHz for hydrogen and 125 MHz for carbon, respectively. The spectra were measured in DMSO-d6 solvent, and chemical shifts are reported as δ values in parts per million (ppm). The infrared spectra were measured by attenuated total reflectance Fourier transform infrared spectroscopy (ATR-FTIR) using a Bruker vacuum spectrometer.

### Zebrafish

Adult *zebrafish* (*D. rerio*), wild-type, both sexes, aged 90–120 days, with sizes of 3.5 ± 0.5 cm and weight 0.4 ± 0.1 g, were obtained from a supplier in Fortaleza (Ceará, Brazil). The animals were acclimatized for 24 h in glass aquariums (30 × 15 × 20 cm^3^) containing dechlorinated water (ProtecPlus) and air pumps with submerged filters, at 25 °C and pH 7.0, with a circadian cycle of 14:10 h light/dark. The fish were anesthetized before drug applications. After the experiments, the fish were sacrificed using cold water (4 °C). All experimental procedures were approved by the Animal Use Ethics Committee of the State University of Ceará (CEUA-UECE), under protocol no. 04983945/2021, in accordance with the Ethical Principles of Animal Experimentation.

### Acute Toxicity 96 h

The adult zebrafish (ZFa, *n* = 6/group) were treated with the synthesized hydrazone compounds (4, 20, and 40 mg/kg; 20 μL; orally). Dimethyl sulfoxide (3% DMSO) was used as a negative control. After 24, 48, 72, and 96 h,^34^ the values obtained from the number of dead zebrafish were subjected to statistical analysis, estimating the lethal dose to kill 50% (LD50) of the zebrafish using the trimmed Spearman-Karber method, with 95% confidence intervals.

### Assessment of Locomotor Activity (Open-Field Test—OFT)

The animals (*n* = 6/group) were treated orally (20 μL; po) with the hydrazones and aldehydes (4, 20, and 40 mg/kg; 20 μL; p.o.). The negative control group (3% DMSO) and positive control group (DZP; 4 mg/kg) were also analyzed. Sixty minutes after the treatments, the animals were placed in glass Petri dishes (10 × 10 cm^2^) containing the same aquarium water, marked with quadrants, and their locomotor activity was analyzed by counting the number of line crossings over a period of 5 min.^35^

### Anxiolytic Assessment (Light/Dark Test—LDT)

To analyze the anxiolytic effect, a glass aquarium (30 cm × 15 cm × 20 cm) with a clear area and a dark area was used, filled with the same aquarium water up to a height of 3 cm. The animals (*n* = 6/group) were treated with hydrazones (HDZI 2,4OH and HDZI 2,3,4OH) and aldehydes (2,4-dihydroxybenzaldehyde and 2,3,4-trihydroxybenzaldehyde) (4, 20, and 40 mg/kg; 20 μL; p.o.). The negative control (3% DMSO) and positive control (DZP 4 mg/kg) groups were also included. Sixty minutes after treatment, the animals were individually placed in the clear zone, and the anxiolytic effect was analyzed based on the time (s) spent in the clear zone of the aquarium during 5 min of observation.^20^

### Assessment of GABAergic Neuromodulation

The GABAergic mechanism of action was investigated using the lowest effective dose. The animals (*n* = 6/group) received a pretreatment with flumazenil (Fmz, 4 mg/kg), a GABAA antagonist, and 15 min later were treated with the lowest effective dose of the HDZI 2,4OH, HDZI 2,3,4OH, 2,4-dihydroxybenzaldehyde, and 2,3,4-trihydroxybenzaldehyde (4 mg/kg; 20 μL; p.o.). Diazepam (DZP, 4 mg/kg; 20 μL; ip) and the negative control (3% DMSO, 20 μL; ip) were also included. Sixty minutes after the treatments, the animals were subjected to the light/dark test, as described in the LDT section.^2,20^

### Assessment of SEROTONergic Neuromodulation

The animals (*n* = 6/group) received a pretreatment with cyproheptadine (cipro, 5-HT2A antagonist, 32 mg/kg, orally), pizotifen (pizo, 5-HT1 and 5-HT2A/2C antagonist), and granisetron (gran, 5-HT3A/3B antagonist, 20 mg/kg, orally).^20^ Thirty minutes later, the samples to be evaluated were administered: fluoxetine (Flx, 0.05 mg/kg, 20 μL, ip), DMSO 3% (20 μL; ip), HDZI 2,4OH, HDZI 2,3,4OH, 2,4-dihydroxybenzaldehyde, and 2,3,4-trihydroxybenzaldehyde (4 mg/kg; 20 μL; v.o). Subsequently, the animal behavior was analyzed in the light/dark test as described in the LDT section.

### MPO-Based Pharmacokinetics Prediction

The chemical structures of HDZI 2,4OH and HDZI 2,3,4OH were plotted in two-dimensional representation using the academic license program MarvinSketch version 23.12, Chemaxon (https://chemaxon.com/marvin), for structural optimization via classical mechanics force field based on the Merck Molecular Force Field (MMFF94) method.^36^ They were also subjected to topological analyses of molecular lipophilicity potential (MLP), topological polar surface area (TPSA), and quantitative estimation of druglikeness using the Central Nervous System Multiparameter Optimization (CNS MPO) algorithm,^37^ as shown in eq 1

1 $$D=\sum_{i=1}^{N}w_{k}T_{k}\left(x_{k}^{0}\right)$$

The order of desirability (*D*) is defined by the weighting factor (*w*) assigned to each physicochemical attribute *k* with a calculated value *x* falling within (*x_k_* ≤ *x*_a_) or outside (*x*_b_ < *x*_k_) the desirability threshold (*T*(*x*)). These attributes include: intrinsic lipophilicity (log *P*) ≤ 3, physiological pH lipophilicity (log *D*7.4) ≤ 2, molecular weight (MW) ≤ 360 g/mol, 75 Å^2^ < TPSA ≤ 120 Å^2^, H-bond donor count (HBD) ≤ 1, and p*K*_a_ most basic ≤ 8 (*N* = 6). This results in a score ranging from 0 to 6 reflecting pharmacokinetic viability.

ADME (absorption, distribution, metabolism, and excretion) attributes were predicted using ADMETlab 2.0 (https://admetmesh.scbdd.com/) and AMDET Prediction Service—Laboratory of Medicinal Chemistry (LMC)—Lomonosov Moscow State University (http://qsar.chem.msu.ru/admet/). These attributes include apparent permeability (Papp, A → B) in the Madin-Darby Canine Kidney (MDCK) cell model, *P*-glycoprotein substrate, intrinsic clearance of unbound fraction in hepatic system (CL_int,u_), volume of distribution (VD), Human Intestinal Absorption (HIA), and blood-brain barrier permeability (BBB).^38^

Prediction of metabolism site was conducted through a test assessing the reactivity degree of molecular fragments or metabolism site for cytochrome P450 (CYP450) isoforms with specificity of structural fragments,^39^ using SOMP Way2Drug (http://www.way2drug.com/somp/), XenoSite (https://xenosite.org/), and STopTox (https://stoptox.mml.unc.edu/) servers.

### Molecular Docking Procedures

The MPO scores were correlated with structure-based virtual screening of target classes using the online server SwissTargetPrediction (http://www.swisstargetprediction.ch/)—Swiss Institute of Bioinformatics. This estimation predicts ligand affinity toward G-protein coupled receptors (GPCRs), ion channels, transporters, and enzymes based on similarity testing with over 370,000 bioactive compounds reported in *Homo sapiens*, *Rattus norvegicus*, and *Mus musculus* organisms, sourced from the ChEMBL database (https://www.ebi.ac.uk/chembl/).^40^

Subsequently, molecular docking simulations were parametrized using the protein structures of the γ-Aminobutyric Acid Type A receptor (GABAA), deposited in the RCSB Protein Data Bank (https://www.rcsb.org/) under code PDB 6HUP, described as “CryoEM structure of human full-length alpha1beta3gamma2L GABA­(A)­R in complex with diazepam (Valium), GABA and megabody Mb38”. This receptor is expressed in *H. sapiens* and *Escherichia coli* and characterized as a membrane protein at a resolution of 3.58 Å by electron microscopy. Additionally, the serotonin receptor Type 3A (5-HT3A) structure was utilized, available in the repository under code PDB 6NP0, described as “Cryo-EM structure of 5-HT3A receptor in the presence of granisetron”. This receptor is expressed in *Spodoptera frugiperda* as a membrane protein at a resolution of 2.92 Å and characterized by electron microscopy.

In AutoDockTools—MGL Tools,^41^ small molecules cocrystallized with the receptors and water molecules (H_2_O) were removed, and Gasteiger charges were added. The grid box for the GABAA receptor was adjusted to coordinates *x* = 125.281, *y* = 139.534, *z* = 136.018 with dimensions *x* = 126, *y* = 100, *z* = 126. For the 5-HT3A receptor, the grid box was set to coordinates *x* = 159.616, *y* = 159.619, *z* = 163.679 with dimensions *x* = 68, *y* = 64, *z* = 122, covering the entire conformational space of the receptors for simulation. Thus, ligands optimized via MMFF94 and receptors adjusted in their conformational spaces were configured for a cycle of 50 independent simulations, each generating 20 poses per ligand. The best-pose selection criteria included an affinity energy < −6.0 kcal/mol, statistical root-mean-square deviation (RMSD) < 2.0 Å, and strength of H-bond interactions calculated from the distance (d) between ligand–receptor.^42^

### Statistical Analysis

Results were expressed as mean values ± standard error for each group of 6 animals. After confirming the normality of distribution and homogeneity of the data, the differences between the groups were identified through the one-way ANOVA in the preliminary tests (OFT and LDT) and two-way ANOVA for the mechanisms of action via GABA and SEROTONinergic, followed by the Tukey test. All analyses were performed using GraphPad Prism v. 8.0. The level of statistical significance was set at 5% (*p* < 0.05).

## Supplementary Material



## Notes

^1^H nuclear magnetic resonance spectrum of HDZI 2,4OH (Figure S1); ^13^C nuclear magnetic resonance spectrum of HDZI 2,4OH (Figure S2); ^1^H nuclear magnetic resonance spectrum of HDZI 2,3,4OH (Figure S3); ^13^C nuclear magnetic resonance spectrum of HDZI 2,3,4OH (Figure S4); FTIR pectrum of HDZI 2,4OH (Figure S5); FTIR spectrum of HDZI 2,3,4OH (Figure S6); GABAergic mechanism of action of HDZI 2,4OH, and HDZI 2,3,4OH, 2,4-dihydroxybenzaldehyde, and 2,3,4-trihydroxybenzaldehyde on the anxiety behavior of adult *zebrafish* in the light and dark test (0–5 min); one-way ANOVA followed by Tukey’s test (Figure S7); SEROTONergic mechanism of action of HDZI 2,4OH, HDZI 2,3,4OH, 2,4-dihydroxybenzaldehyde, and 2,3,4-trihydroxybenzaldehyde (CIPRO—cyproheptadine, PIZO—pizotifen, GRAN—granisetron) on the anxiety behavior of adult zebrafish in the light and dark test (0–5 min) (Figure S8); molecular lipophilicity potential (MLP) surface map plotted for the compounds HDZI 2,4OH, HDZI 2,3,4OH, 2,4-dihydroxybenzaldehyde, 2,3,4-trihydroxybenzaldehyde, and ISOZD; druglikeness radar plot generated by Pfizer’s MPO algorithm (Figure S9); alignment between lipophilicity (log *P*) and topological polar surface area (TPSA) for estimation of CNS safety, alignment between molecular weight (MW) and lipophilicity at pH 7.4 (log *D*) for estimation of cellular permeability (Papp) and hepatic clearance (CL_int,u_), prediction of human intestinal absorption (HIA), logarithm of blood-brain barrier permeability (logBB) of the compounds HDZI 2,4OH and HDZI 2,3,4OH, and prediction of HIA and (f) logBB of the precursors 2,4-dihydroxybenzaldehyde and 2,3,4-trihydroxybenzaldehyde (Figure S10); site of metabolism prediction of HDZI 2,4OH, HDZI 2,3,4OH, 2,4-dihydroxybenzaldehyde, 2,3,4-trihydroxybenzaldehyde and ISOZD, where green colors indicate safer fragments (low reactivity) and red colors represent toxic fragments (Figure S11) (PDF)

## References

[1] Salpekar J. A., Basu T., Thangaraj S., Maguire J. (2020). The intersections of
stress, anxiety and epilepsy. Res. Neurosci..

[2] Biney R. P., Benneh C. K., Kyekyeku J. O., Ameyaw E. O., Boakye-Gyasi E., Woode E. (2018). Attenuation of anxiety
behaviours by xylopic acid in mice and zebrafish
models of anxiety disorder. Pharm. Biosci. J..

[3] Graf H., Walter M., Metzger C. D., Abler B. (2014). Antidepressant-Related
Sexual Dysfunction — Perspectives from Neuroimaging. Pharm. Biochem. Behav..

[4] Hood S. D., Norman A., Hince D. A., Melichar J. K., Hulse G. K. (2014). Benzodiazepine
Dependence and Its Treatment with Low Dose Flumazenil. Br. J. Clin. Pharmacol..

[5] Harro J. (2018). Animals, anxiety,
and anxiety disorders: How to measure anxiety in rodents and why. Behav. Brain Res..

[6] Cassar S., Adatto I., Freeman J. L., Gamse J. T., Iturria I., Lawrence C., Muriana A., Peterson R. T., Van Cruchten S., Zon L. I. (2020). Use of Zebrafish in Drug Discovery
Toxicology. Chem. Res. Toxicol..

[7] da
Silva A. W., Ferreira M. K. A., Pereira L. R., Reboucas E. L., Coutinho M. A., Dos J., Lima J. R., Guedes M. I. F., Bandeira P. N., de Menezes J. E. S. A., Marinho M. M., Teixeira A. M. R., Trevisan M. T. S., dos Santos H. S., Marinho E. S. (2022). Combretum lanceolatum extract reverses anxiety and
seizure behavior in adult zebrafish through GABAergic neurotransmission:
an in vivo and in silico study. J. Biomol. Struct.
Dyn..

[8] Ferreira M. K. A., Silva A. W., Moura A. L. S., Sales K. V. B., Marinho E. M., Cardoso J. N. M., Marinho M. M., Bandeira P. N., Magalhaes F. E. A., Marinho E. S., Menezes J. E. S. A., Santos H. S. (2021). Chalcones reverse
the anxiety and convulsive behavior of adult zebrafish. Epilepsy Behav..

[9] Arruda I. E., Macedo B. V. S., Macedo J. C., Campos W. R. A., Araújo C. R. M., Gonsalves A. A. (2020). Preparação
de hidrazona e N-acilidrazone
usando fármacos comerciais como reagentes: aulas práticas
de síntese de compostos bioativos. Quím.
Nova.

[10] Ragab M. A., Eldehna W. M., Nocentini A., Bonardi A., Okda H. E., Elgendy B., Ibrahim T. S., Abd-Alhaseeb M. M., Gratteri P., Supuran C. T., Al-Karmalawy A. A., Elagawany M. (2023). 4-(5-Amino-pyrazol-1-yl) benzenesulfonamide derivatives
as novel multi-target anti-inflammatory agents endowed with inhibitory
activity against COX-2, 5-LOX and carbonic anhydrase: design, synthesis,
and biological assessments. Eur. J. Med. Chem..

[11] Udumula M. P., Bhat A., Mangali S., Kalra J., Dhar I., Srirama D., Dhara A. (2018). Pharmacological
evaluation of novel
PKR inhibitor indirubin-3-hydrazone in-vitro in cardiac myocytes and
in-vivo in Wistar rats. Life Sci..

[12] De
Souza M. A., De Castro K. K. A., Almeida-Neto F. W. Q., Bandeira P. N., Ferreira M. K. A., Marinho M. M., Da Rocha M. N., De Brito D. H. A., Mendes F. R. M., Rodrigues T. H. S., De Oliveira M. R., De Menezes J. E. S. A., Barreto A. C. H., Marinho E. S., Lima-Neto P., Dos Santos H. S., Teixeira A. M. R. (2022). Structural and spectroscopic analysis,
ADMET study, and anxiolytic-like effect in adult zebrafish (*Danio rerio*) of 4′-[(1E, 2E)-1-(2-(2′,
4′-dinitrophenyl) hydrazone-3-(4-methoxyphenyl) allyl) aniline. J. Mol. Struct..

[13] Mokhnache K., Karbab A., Soltani E. K., Bououden W., Ouhida S., Arrar L., Esteban M. A., Charef N., Mubarak M. S. (2020). Synthesis,
characterization, toxic substructure prediction, hepatotoxicity evaluation,
marine pathogenic bacteria inhibition, and DFT calculations of a new
hydrazone derived from isoniazid. J. Mol. Struct..

[14] Tal T., Yaghoobi B., Lein P. J. (2020). Translational
toxicology in zebrafish. Curr. Opin. Toxicol..

[15] Popiołek Ł., Piątkowska-Chmiel I., Gawrońska-Grzywacz M., Biernasiuk A., Izdebska M., Herbet M., Sysa M., Malm A., Dudka J., Wujec M. (2018). New hydrazide-hydrazones
and 1,3-thiazolidin-4-ones with 3-hydroxy-2-naphthoic moiety: Synthesis,
in vitro and in vivo studies. Biomed. Pharmacother..

[16] Gebauer D. L., Pagnussat N., Piato A. L., Schaefer I. C., Bonan C. D., Lara D. R. (2011). Effects
of anxiolytics in zebrafish: similarities and
differences between benzodiazepines, buspirone and ethanol. Pharm. Biochem. Behav..

[17] Resende, R. R. ; Soccol, C. R. Capítulo 1: Zebrafish Como Modelo Para Estudos Comportamentais. In Biotecnologia Aplicada à Saúde: Fundamentos e Aplicações, 1st ed.; Editora Blucher, 2015.

[18] Cachat J., Stewart A., Utterback E., Hart P., Gaikwad S., Wong K., Kyzar E., Wu N., Kalueff A. V. (2011). Three-Dimensional
Neurophenotyping of Adult Zebrafish Behavior. PLoS One.

[19] Gupta P., Khobragade S. B., Shingatgeri V., Rajaram S. (2014). Assessment of locomotion
behavior in adult Zebrafish after acute exposure to different pharmacological
reference compounds. Drug Dev. Ther..

[20] Benneh C. K., Biney R. P., Mante P. K., Tandoh A., Adongo D. W., Woode E. (2017). Maerua angolensis stem
bark extract reverses anxiety and related
behaviours in zebrafish—Involvement of GABAergic and 5-HT systems. J. Ethnopharmacol..

[21] Mansur B. M., Santos B. R., Gouveia A. (2014). Efeitos da Substância
de Alarme no Teste Claro/Escuro no Zebrafish, *Danio
rerio*. Biota Amazônia.

[22] Cao Y., Yan H., Yu G., Su R. (2019). Flumazenil-insensitive benzodiazepine
binding sites in GABAA receptors contribute to benzodiazepine-induced
immobility in zebrafish larvae. Life Sci..

[23] Higgs J., Wasowski C., Marcos A., Jukić M., Pavan C. H., Gobec S., De Tezanos
Pinto F., Colettis N., Marder M. (2019). Chalcone derivatives:
synthesis,
in vitro and in vivo evaluation of their anti-anxiety, anti-depression
and analgesic effects. Heliyon.

[24] Wager T. T., Hou X., Verhoest P. R., Villalobos A. (2016). Central Nervous System Multiparameter
Optimization Desirability: Application in Drug Discovery. ACS Chem. Neurosci..

[25] Kiani Y. S., Jabeen I. (2020). Lipophilic Metabolic Efficiency (LipMetE)
and Drug
Efficiency Indices to Explore the Metabolic Properties of the Substrates
of Selected Cytochrome P450 Isoforms. ACS Omega.

[26] Hughes T. B., Miller G. P., Swamidass S. J. (2015). Modeling
Epoxidation of Drug-like
Molecules with a Deep Machine Learning Network. ACS Cent. Sci..

[27] Johnson T. W., Dress K. R., Edwards M. (2009). Using the Golden Triangle
to optimize
clearance and oral absorption. Bioorg. Med.
Chem. Lett..

[28] Radchenko E. V., Dyabina A. S., Palyulin V. A., Zefirov N. S. (2016). Prediction of human
intestinal absorption of drug compounds. Russ.
Chem. Bull..

[29] Dyabina A. S., Radchenko E. V., Palyulin V. A., Zefirov N. S. (2016). Prediction of blood-brain
barrier permeability of organic compounds. Dokl.
Biochem. Biophys..

[30] Pires, D. E. V. ; Kaminskas, L. M. ; Ascher, D. B. Prediction and Optimization of Pharmacokinetic and Toxicity Properties of the Ligand. In Computational Drug Discovery and Design, Methods in Molecular Biology; Springer, 2018; Vol. 1762, pp 271–284 10.1007/978-1-4939-7756-7_14. 29594777

[31] Marinho E. M., Andrade-Neto J. B., Silva J., Silva C. R., Cavalcanti B. C., Marinho E. S., Nobre-Junior H. V. (2020). Virtual
screening based on molecular
docking of possible inhibitors of Covid-19 main protease. Microb. Pathogen..

[32] Masiulis S., Desai R., Uchański T., Serna Martin I., Laverty D., Karia D., Malinauskas T., Zivanov J., Pardon E., Kotecha A., Steyaert J., Miller K. W., Aricescu A. R. (2019). GABAA receptor signalling mechanisms
revealed by structural pharmacology. Nature.

[33] Imberty A., Hardman K. D., Carver J. P., Perez S. (1991). Molecular modelling
of protein-carbohydrate interactions. Docking of monosaccharides in
the binding site of concanavalin A. Glycobiology.

[34] OECD . OECD Guidelines for the Testing of Chemicals. [s.l: s.n.]­p. No.203. 1992.

[35] Guedes J. M., Ferreira M. K. A., Oliveira L. S., Silva A. W., Menezes J. E. S. A., Bandeira P. N., Teixeira A. M. R., Marinho E. S., Marinho M. M., Santos H. S. (2023). Anxiolytic-Like
Effect In Adult Zebrafish (*Danio rerio*) Through Gabaergic System And Molecular
Docking Study Of Chalcone (E)-1-(2-Hydroxy-3,4,6-Trimethoxyphenyl)-3-(4-Methoxyphenyl)­Prop-2-En-1-One. Biointerface Res. Appl. Chem..

[36] Halgren T. A. (1996). Merck molecular
force field. I. Basis, form, scope, parameterization, and performance
of MMFF94. J. Comput. Chem..

[37] Wager T. T., Hou X., Verhoest P. R., Villalobos A. (2010). Moving beyond
Rules: The Development
of a Central Nervous System Multiparameter Optimization (CNS MPO)
Approach To Enable Alignment of Druglike Properties. ACS Chem. Neurosci..

[38] Da
Rocha M. N., da Fonseca A. M., Dantas A. N. M., dos
Santos H. S., Marinho E. S., Marinho G. S. (2024). In Silico Study
in MPO and Molecular Docking of the Synthetic Drynaran Analogues Against
the Chronic Tinnitus: Modulation of the M1Muscarinic Acetylcholine *Receptor*. Mol. Biotechnol..

[39] Zheng M., Luo X., Shen Q., Wang Y., Du Y., Zhu W., Jiang H. (2009). Site of metabolism
prediction for six biotransformations mediated
by cytochromes P450. Bioinformatics.

[40] Daina A., Michielin O., Zoete V. (2019). SwissTargetPrediction: updated data
and new features for efficient prediction of protein targets of small
molecules. Nucleic Acids Res..

[41] Morris G. M., Huey R., Lindstrom W., Sanner M. F., Belew R. K., Goodsell D. S., Olson A. J. (2009). AutoDock4 and AutoDockTools4: Automated
docking with selective receptor flexibility. J. Comput. Chem..

[42] Marinho E. M., De Andrade Neto J. B., Silva J., Da Silva C. R., Cavalcanti B. C., Marinho E. S., Nobre Júnior H.
V. (2020). Virtual screening based
on molecular docking of possible inhibitors of Covid-19 main protease. Microb. Pathogen..

[43] Finberg J. P. M., Rabey J. M. (2016). Inhibitors of MAO-A
and MAO-B in psychiatry and neurology. Front.
Pharmacol..

[44] Youdim M. B. H., Edmondson D., Tipton K. F. (2006). The therapeutic potential of monoamine
oxidase inhibitors. Nat. Rev. Neurosci..

