# Supplementary material for: Synthesis, Characterization, and Evaluation of the Anxiolytic Activity of Hydrazones Derived from the Drug Isoniazid, Using the Adult Zebrafish (Danio rerio) Model
Source: ACS Omega. 2025 Sep 25;10(39):45152–64. doi: 10.1021/acsomega.5c04279 (PMC12508942; doi:10.1021/acsomega.5c04279)
Supplement: Supplementary file 1 [file ao5c04279_si_001.pdf]

SYNTHESIS, CHARACTERIZATION, AND EVALUATION OF THE ANXIOLYTIC ACTIVITY OF HYDRAZONES DERIVED FROM THE DRUG ISONIAZID, USING THE ADULT ZEBRAFISH (DANIO RERIO) MODEL

Amanda Maria Barros Alves[a], Ivana Carneiro Romão[a], Rhadija Jorge Souza[a], Emmanuel Silva Marinho[a], Márcia Machado Marinho[a], Matheus Nunes Rocha[a], Kirley Marques Canuto[b], Jane Eire Silva Alencar Menezes[a], Sônia Maria Costa Siqueira[a], Hélcio Silva dos Santos[a*]

[a] Universidade Estadual do Ceará, Programa de Pós-Graduação em Ciências Naturais, Fortaleza, Ceará 60714-903, Brasil

[b] Empresa Brasileira de Pesquisa Agropecuária, Embrapa, Fortaleza, Ceará 60511-110, Brasil.

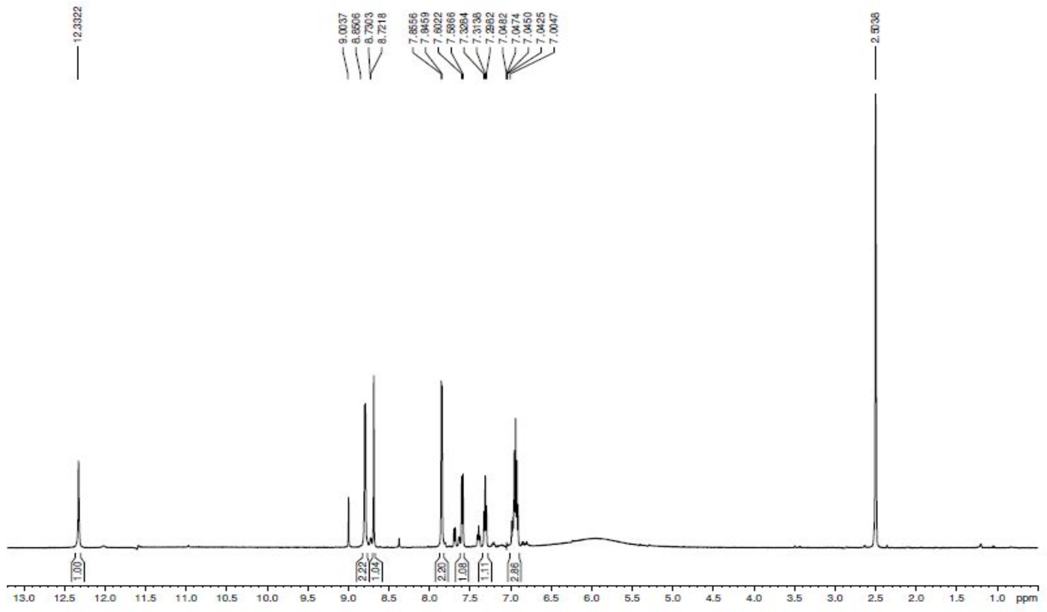

Figure S1– ^{1}H Nuclear Magnetic Resonance Spectrum of HDZI 2,4OH

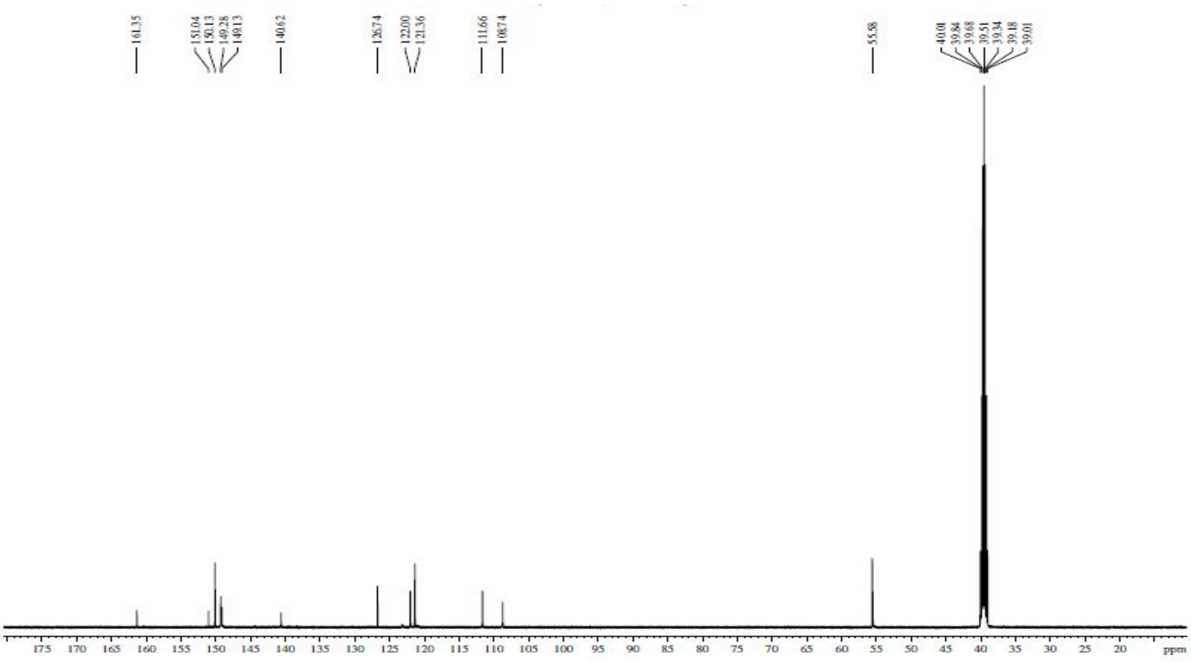

Figure S2 – ^{13}C Nuclear Magnetic Resonance Spectrum of HDZI 2,4OH

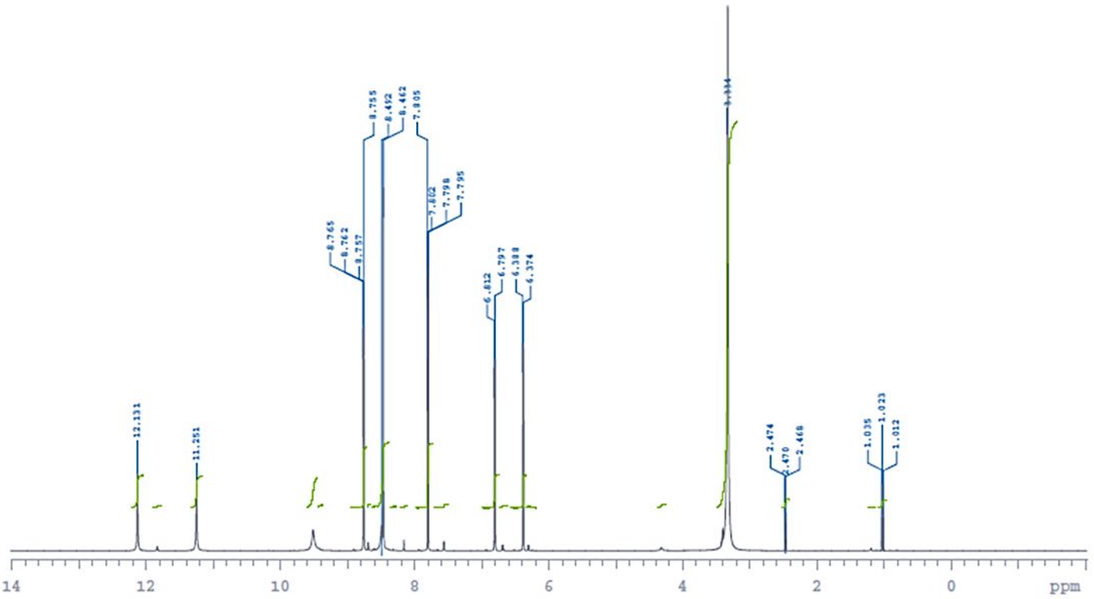

Figure S3 – [1]H Nuclear Magnetic Resonance Spectrum of HDZI 2,3,4OH

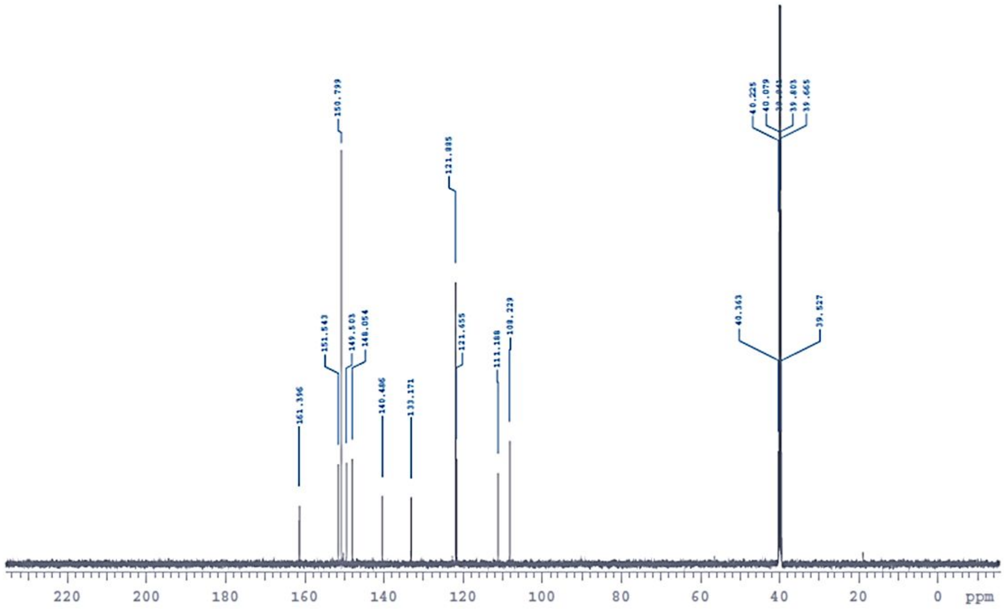

Figure S4 – [13]C Nuclear Magnetic Resonance Spectrum of HDZI 2,3,4OH

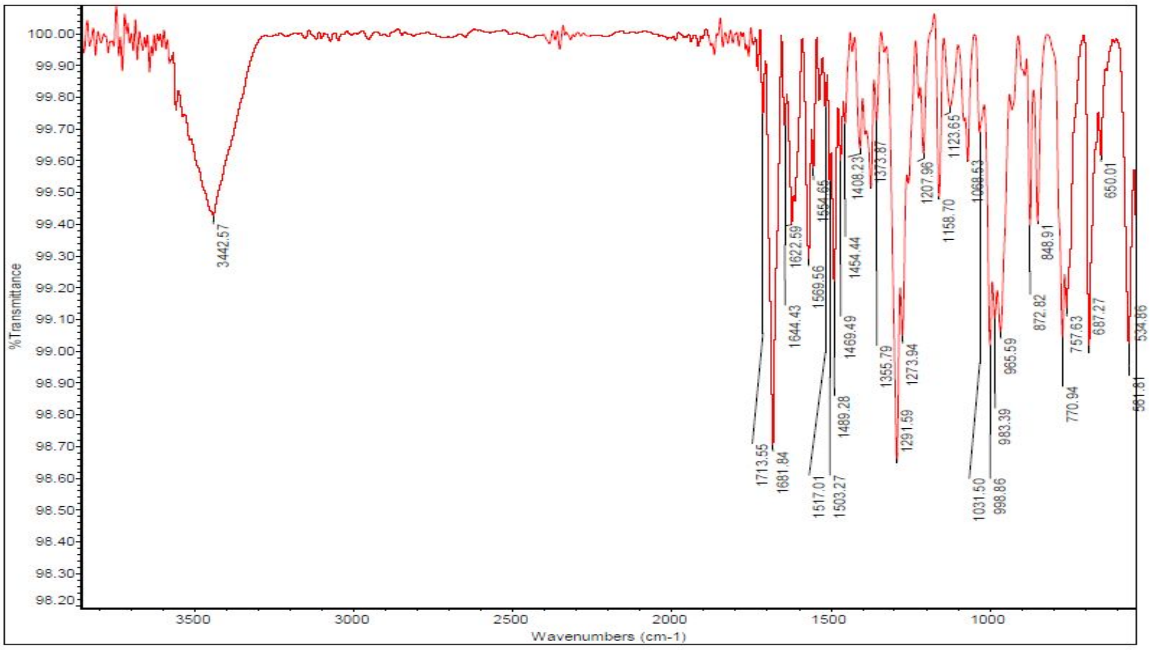

Figure S5 – FTIR Spectrum of HDZI 2,4OH

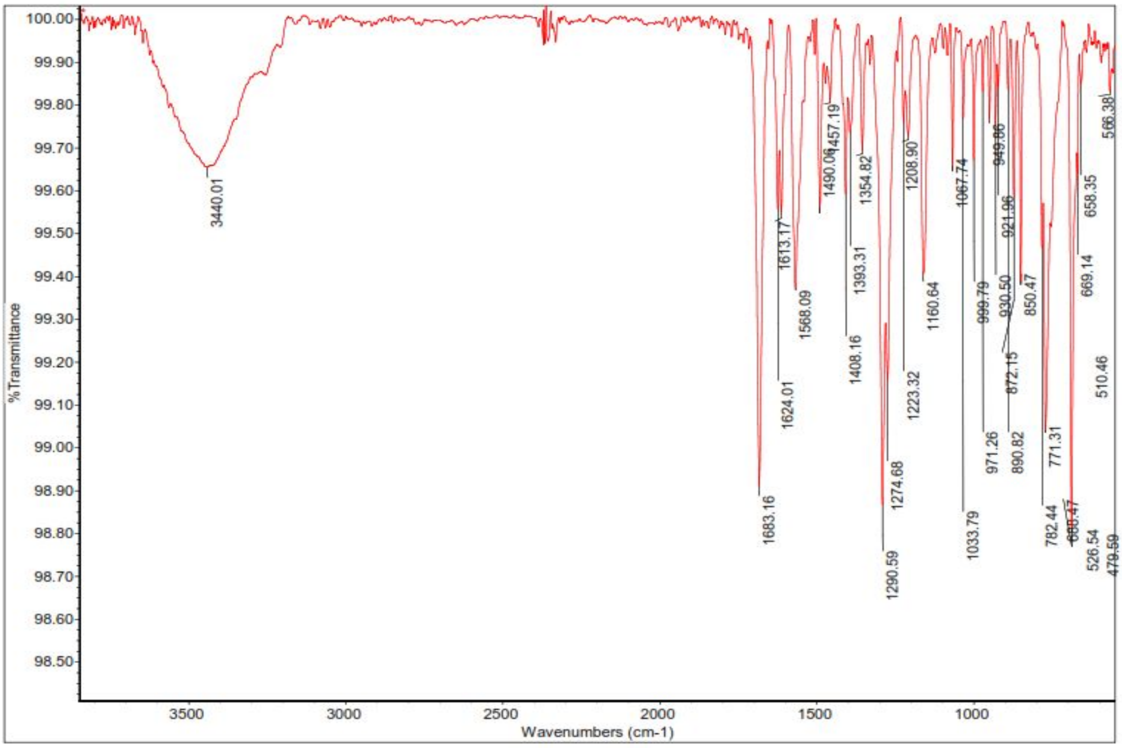

Figure S6 – FTIR Spectrum of HDZI 2,3,4OH

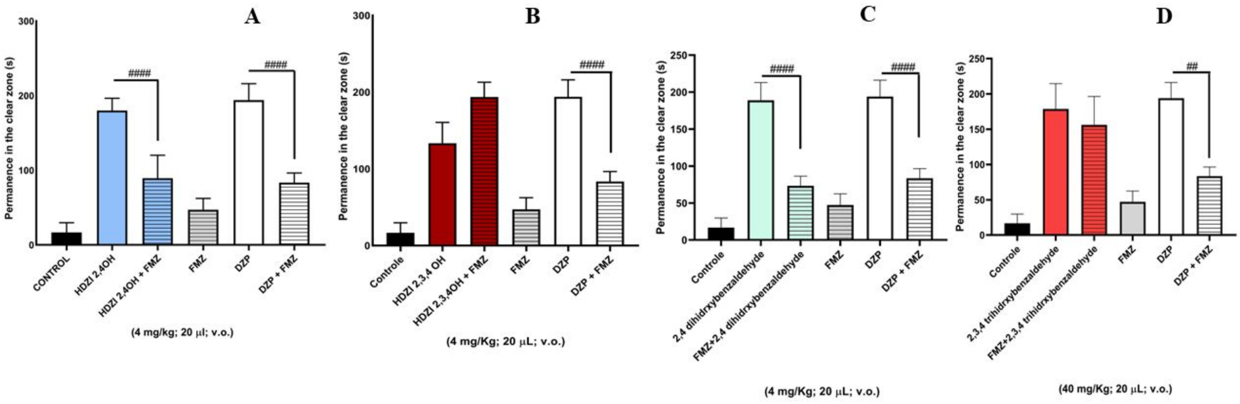

Figure S7 – GABAergic mechanism of action of HDZI 2,4OH (A) and HDZI 2,3,4OH (B), 2,4 dihidroxybendaldehyde (C) and 2,3,4 trihidroxybenzaldehyde (D) on the anxiety behavior of adult *zebrafish* in the light and dark test (0–5 min). Each column represents the mean ± standard error of the mean. One-Way ANOVA followed by Tukey's test.

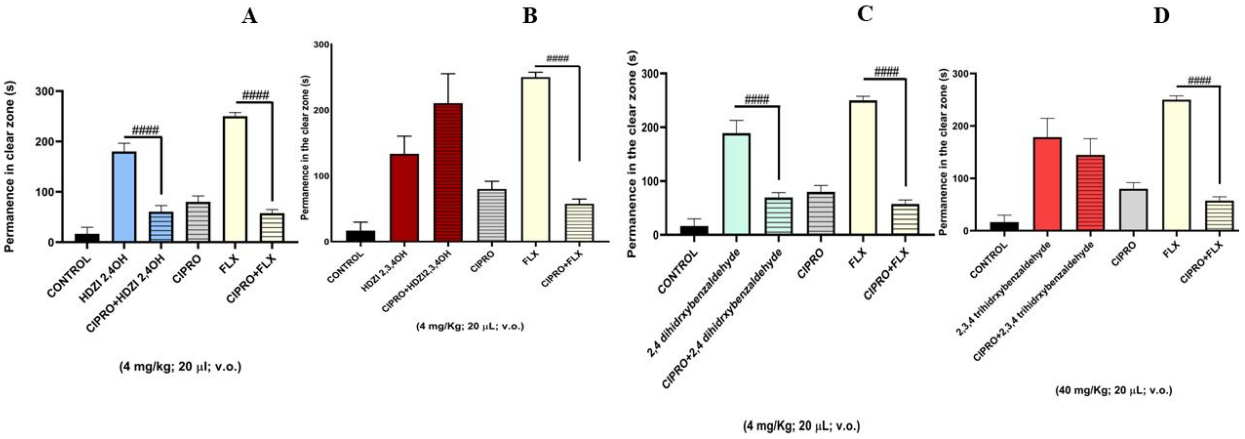

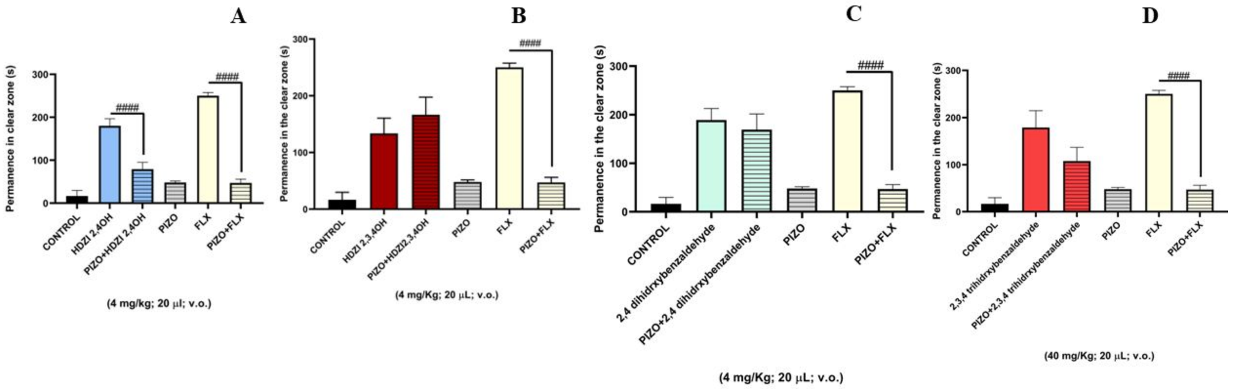

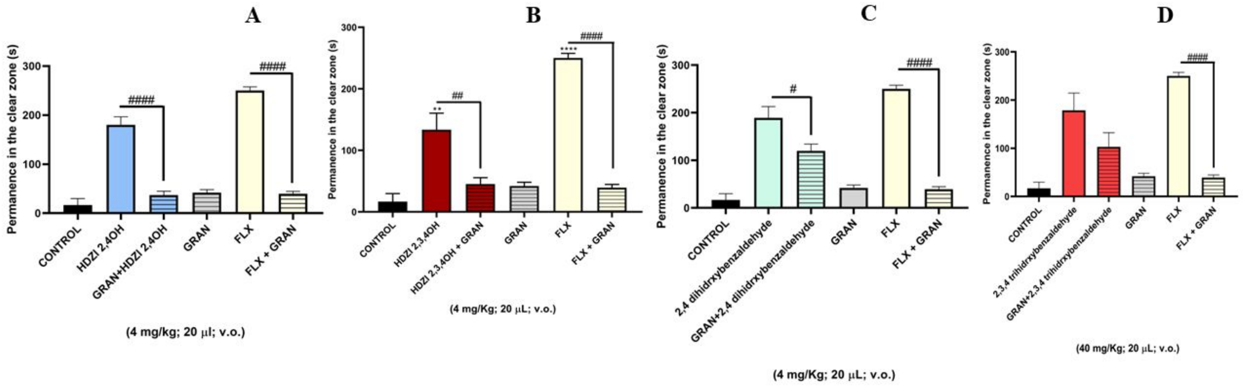

Figure S8 – SEROTONergic mechanism of action of HDZI 2,4OH (A) and HDZI 2,3,4OH (B), 2,4 dihidroxybendaldehyde (C) and 2,3,4 trihidroxybenzaldehyde (D) (CIPRO - cyproheptadine, PIZO - pizotifen, GRAN - granisetron) on the anxiety behavior of adult zebrafish in the light and dark test (0–5 min).

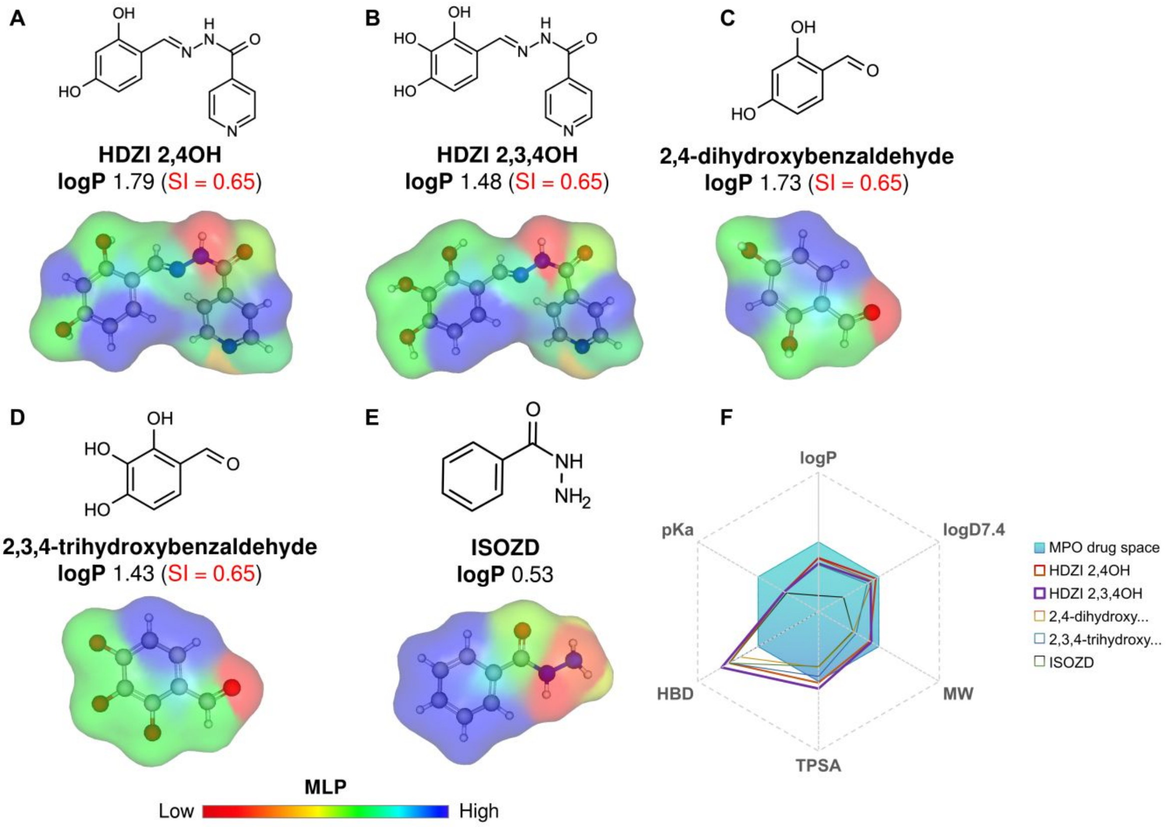

Figure S9 – Molecular lipophilicity potential (MLP) surface map plotted for the compounds (a) HDZI 2,4OH, (b) HDZI 2,3,4OH, (c) 2,4-dihydroxybenzaldehyde, (d) 2,3,4-trihydroxybenzaldehyde, and (e) ISOZD. (F) Druglikeness radar plot generated by Pfizer's MPO algorithm.

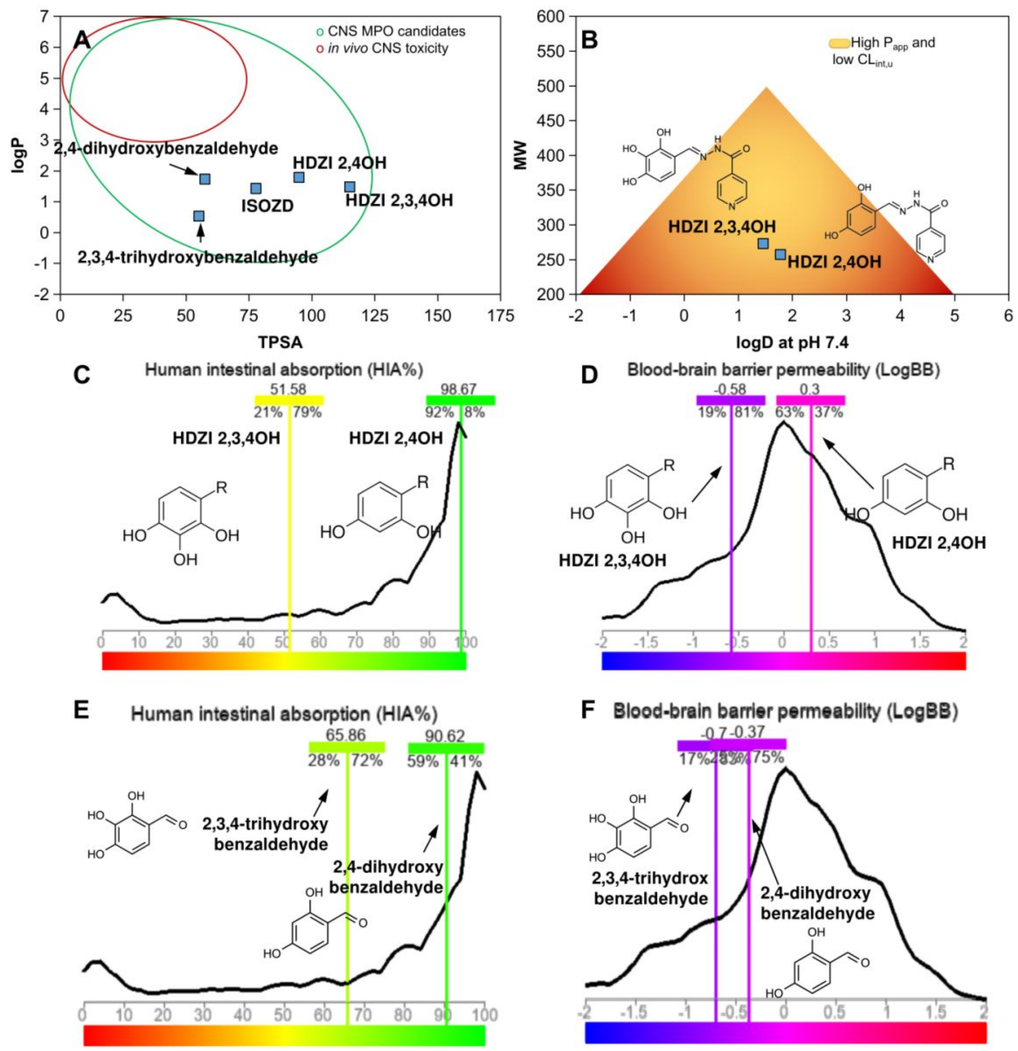

Figure **S10 –** (a) Alignment between lipophilicity (logP) and topological polar surface area (TPSA) for estimation of CNS safety, (b) alignment between molecular weight (MW) and lipophilicity at pH 7.4 (logD) for estimation of cellular permeability (Papp) and hepatic clearance (CLint,u). (c) Prediction of human intestinal absorption (HIA) and (d) logarithm of blood-brain barrier permeability (logBB) of the compounds HDZI 2,4OH and HDZI 2,3,4OH, (e) prediction of HIA and (f) logBB of the precursors 2,4-dihydroxybenzaldehyde and 2,3,4-trihydroxybenzaldehyde.

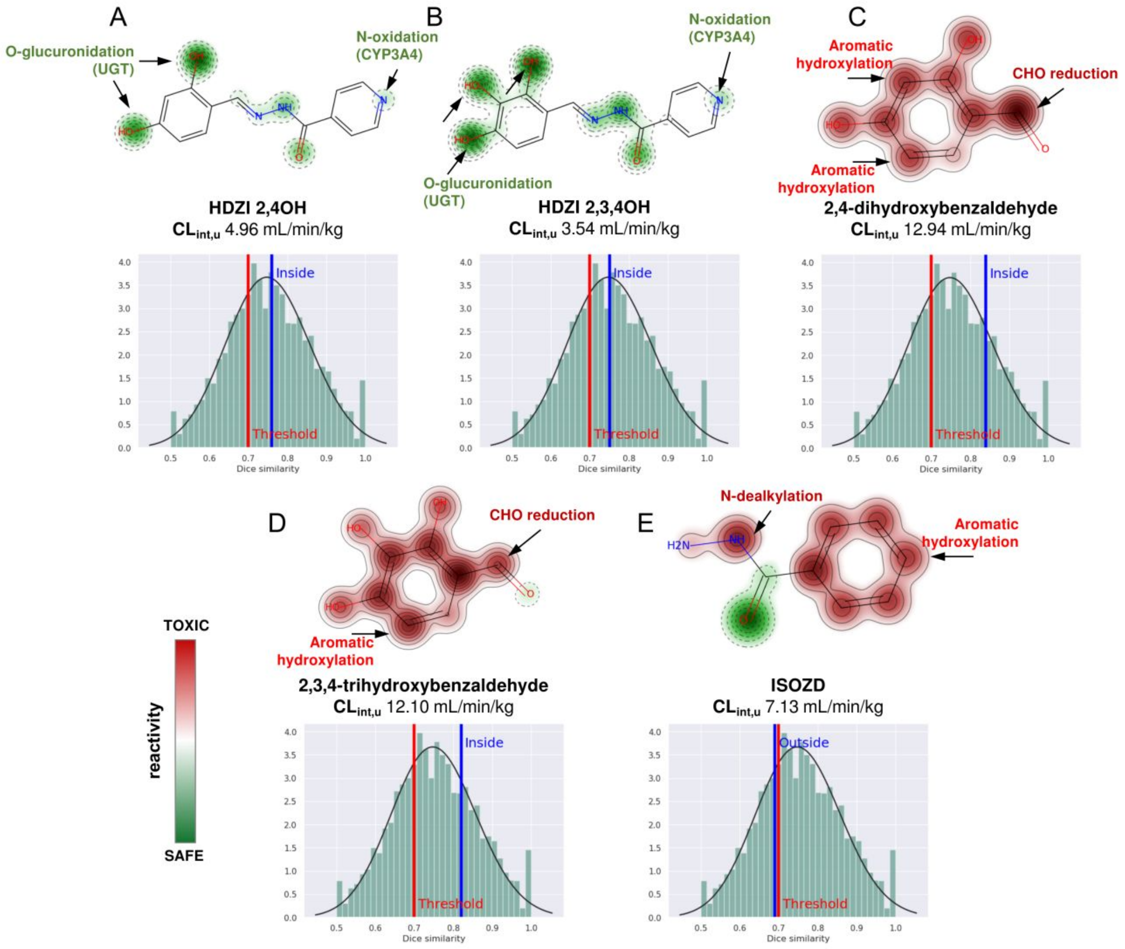

Figure S11 – Site of metabolism prediction of (a) HDZI 2,4OH, (b) HDZI 2,3,4OH, (c) 2,4-dihidroxibenzaldeído, (d) 2,3,4-trihidroxibenzaldeído and (e) ISOZD, where green colors indicate safer fragments (low reactivity) and red colors represent toxic fragments.
